# A systematic study on the integration of MRI connectivity metrics for Alzheimer's diagnosis, staging, and cognitive decline prediction

**DOI:** 10.3389/fnimg.2026.1746464

**Published:** 2026-02-17

**Authors:** Shahzad Ali, Wendy Kreshpa, Nicola Rosso, Michele Piana, Luca Roccatagliata, Alessio Cirone, Lorenzini Luigi, Cristina Campi, Matteo Pardini, Sara Garbarino

**Affiliations:** 1 Department of Pharmacy and Biotechnology, Alma Mater Studiorum - Universitá di Bologna, Bologna, Italy; 2 Life Science Computational Laboratory (LISCOMP), IRCCS Ospedale Policlinico San Martino, Genova, Italy; 3 Department of Information Sciences, University of Education, Lahore, Pakistan; 4 IRCCS Ospedale Policlinico San Martino, Genova, Italy; 5 Dipartimento di Neuroscienze, riabilitazione, oftalmologia, genetica e scienze materno-infantili, Universitá degli Studi di Genova, Genova, Italy; 6 Dipartimento di Matematica, Universitá degli Studi di Genova, Genova, Italy

**Keywords:** Alzheimer's disease, ensemble learning, graph theory metrics, machine learning, MRI

## Abstract

Alzheimer's disease (AD) is a degenerative neurological disorder marked by cognitive decline and functional disability. Despite the extensive use of magnetic resonance imaging (MRI) in machine learning (ML)-based AD studies, the relative and combined contributions of MRI-derived morphometric (MO), microstructural (MS), and graph-theoretical (GT) features are still not well explored in a unified, comparative framework. It remains unclear whether adding multimodal MRI-derived features consistently improves the predictive performance of ML-based approaches for AD diagnosis and cognitive decline. Addressing this gap, this study systematically analyzed the individual (MO, MS, GT) and combined (MO+MS, MO+GT, MS+GT, MO+MS+GT) utility of MRI-based feature sets. We developed an ensemble-based ML framework with a nested cross-validation module for two key tasks: (i) Alzheimer's disease cognitive stage classification (DSC) and (ii) longitudinal cognitive decline prediction (LCDP) in terms of mini-mental state examination (MMSE) score. In this study, we conducted feature ablation and statistical analysis to evaluate performance improvements resulting from the incremental addition of feature sets. The results of the study indicated that the proposed ensemble-based ML approach achieved the best predictive performance (balanced accuracy [BACC]: 0.898 ± 0.051) using a combination of MO and MS feature sets for cognitively normal (CN) vs. AD dementia (CN–ADD). In contrast, the best results for mild cognitive impairment (MCI) vs. ADD (MCI–ADD) and CN–MCI were achieved using the MO feature set alone, with BACC of 0.769 ± 0.116 and 0.652 ± 0.044, respectively. Likewise, for the LCDP task, the MO-based ensemble learner achieved an R^2^ of 0.212 ± 0.177. These results demonstrate that MO features capture the most robust disease-related information, while multimodal integration offers task-specific and limited benefits. In addition, these findings demonstrate the potential of integrated MRI-derived features in ML frameworks for enhancing ADD diagnosis and cognitive decline prediction and underscore the importance of feature selection based on task complexity.

## Highlights

- Systematic integration of multimodal MRI-derived metrics for Alzheimer's diagnosis and staging.
- Identification of significant metric–region associations linked to disease progression.
- Enhanced explainability and interpretability through SHAP and LIME analyses.
- Demonstrated potential for early detection and cognitive decline prediction in Alzheimer's disease.

## 1 Introduction

Alzheimer's disease (AD) is the most common form of progressive dementia, affecting millions worldwide and posing a major public health challenge (Fan et al., 2022). It typically begins with memory loss, language difficulties, and cognitive impairments, ultimately leading to the loss of functional independence (Zhang et al., 2023; Liu et al., 2023). Clinically, AD is commonly viewed as a cognitive continuum, progressing from cognitively normal (CN) aging through mild cognitive impairment (MCI) to Alzheimer's disease dementia (ADD) (Aisen et al., 2017). The underlying pathology of ADD includes misfolded extracellular amyloid-β and intracellular hyperphosphorylated tau, which lead to neurodegeneration, synaptic loss, and macroscopic brain atrophy (Lane et al., 2018). While *in vivo* biomarkers targeting amyloid-β and tau have been developed and are clinically used to aid diagnosis, their role in helping capture the severity of functional cognitive decline (i.e., their correlation with cognitive performance and the progression of individual subjects along the continuum from normal cognition to dementia) remains a challenge. Consequently, there is growing interest in accurate early-stage diagnosis and in identifying robust, generalizable biomarkers that can support timely intervention, which may help slow disease progression and improve outcomes (Liu et al., 2023; Robinson et al., 2015).

To address this need, advances in neuroimaging combined with machine learning (ML) have enabled quantitative characterization of brain changes associated with ADD. Structural MRI (sMRI)–derived morphometric (MO) features quantify regional brain atrophy (i.e., brain volume, cortical thickness, etc.) (Tae et al., 2025), whereas diffusion MRI (dMRI) provides complementary information by capturing microstructural (MS) white matter alterations that may precede overt atrophy (Henriques et al., 2023). In addition to MO and MS features, graph-theoretical (GT) metrics derived from neuroimaging data (such as dMRI) enable characterization of large-scale brain network organization and its alterations across neurological and psychiatric conditions (Phillips et al., 2015; Madole et al., 2023). Several studies have used ML approaches in recent years to analyze MRI-derived features for the characterization of Alzheimer's disease–related brain changes and cognitive impairment. For example, Dyrba et al. (2013) applied ML to dMRI-derived MS features, including fractional anisotropy (FA) and mean diffusivity (MD), to classify Alzheimer's disease and cognitively normal subjects and demonstrated the sensitivity of MS white-matter alterations to Alzheimer's disease. Ebadi et al. (2017) employed an ensemble ML framework using GT metrics extracted from dMRI-based structural connectivity networks to distinguish ADD and MCI, highlighting the contribution of network-level properties (i.e., degree and closeness centrality in regions such as the somatosensory association and primary sensory cortices). Similarly, Karim et al. (2024) used GT metrics derived from resting-state functional MRI (rs-fMRI)-based connectivity networks to identify disease-related network-level disruptions. In contrast, Diogo et al. (2022) combined sMRI-derived MO features with GT metrics and reported that MO features, particularly hippocampal measures, dominated classification performance, while GT features did not consistently improve accuracy. Collectively, these studies highlight the utility of individual MRI-derived feature representations in Alzheimer's disease research.

Despite these advances, it remains unclear whether adding multimodal MRI-derived features consistently improves the predictive performance of ML-based approaches for AD diagnosis and cognitive decline. Most prior studies have typically examined MRI-derived feature representations either in isolation (Dyrba et al., 2013; Ebadi et al., 2017) or in limited combinations (Diogo et al., 2022). Even when multiple feature types are considered, their relative and incremental contributions are rarely assessed systematically, making it challenging to determine whether increased feature complexity yields consistent performance gains. For example, diffusion MRI–based studies such as Dyrba et al. (2013) focused exclusively on MS features, while Ebadi et al. (2017) and Karim et al. (2024) evaluated GT metrics derived from structural and functional connectivity networks, respectively, without comparison to MO or MS feature representations. Although global network properties (e.g., small-worldness) have been widely studied, the contribution of local network dynamics to regional connectivity remains less explored (Ebadi et al., 2017). Moreover, most existing studies emphasize cross-sectional diagnostic classification and provide limited insight into longitudinal cognitive decline or model interpretability. Consequently, it remains unclear when, and for which tasks, combining different MRI-derived feature representations is beneficial. Collectively, these limitations highlight the need for a systematic and interpretable evaluation of MRI-derived feature representations, both individually and incrementally, and for extending analysis beyond cross-sectional classification to include markers of cognitive decline.

To address these limitations, we propose a rigorous ML framework that systematically evaluates the predictive utility and interpretability of diverse MRI-derived feature representations. Using data from the Alzheimer's Disease Neuroimaging Initiative (ADNI) (Jack Jr et al., 2008), we systematically evaluate MO, MS, and GT features both individually and in combination for two complementary tasks: Alzheimer's disease cognitive stage classification (DSC) and longitudinal cognitive decline prediction (LCDP), quantified using changes in Mini-Mental State Examination (MMSE) scores over time. In addition, the proposed framework incorporates explainable artificial intelligence (XAI) techniques, such as SHapley Additive exPlanations (SHAP) (Salih et al., 2025) and Local Interpretable Model-Agnostic Explanations (LIME) (Ribeiro et al., 2016), to explicitly address the interpretability challenge associated with high-dimensional feature spaces, which enables transparent assessment and interpretation of the contributions of individual features to model predictions. In summary, the proposed systematic and interpretable ML framework aims to determine when increased feature complexity improves model performance and to identify MRI-derived features relevant to disease staging and cognitive decline.

## 2 Materials and methods

**Figure 1:**
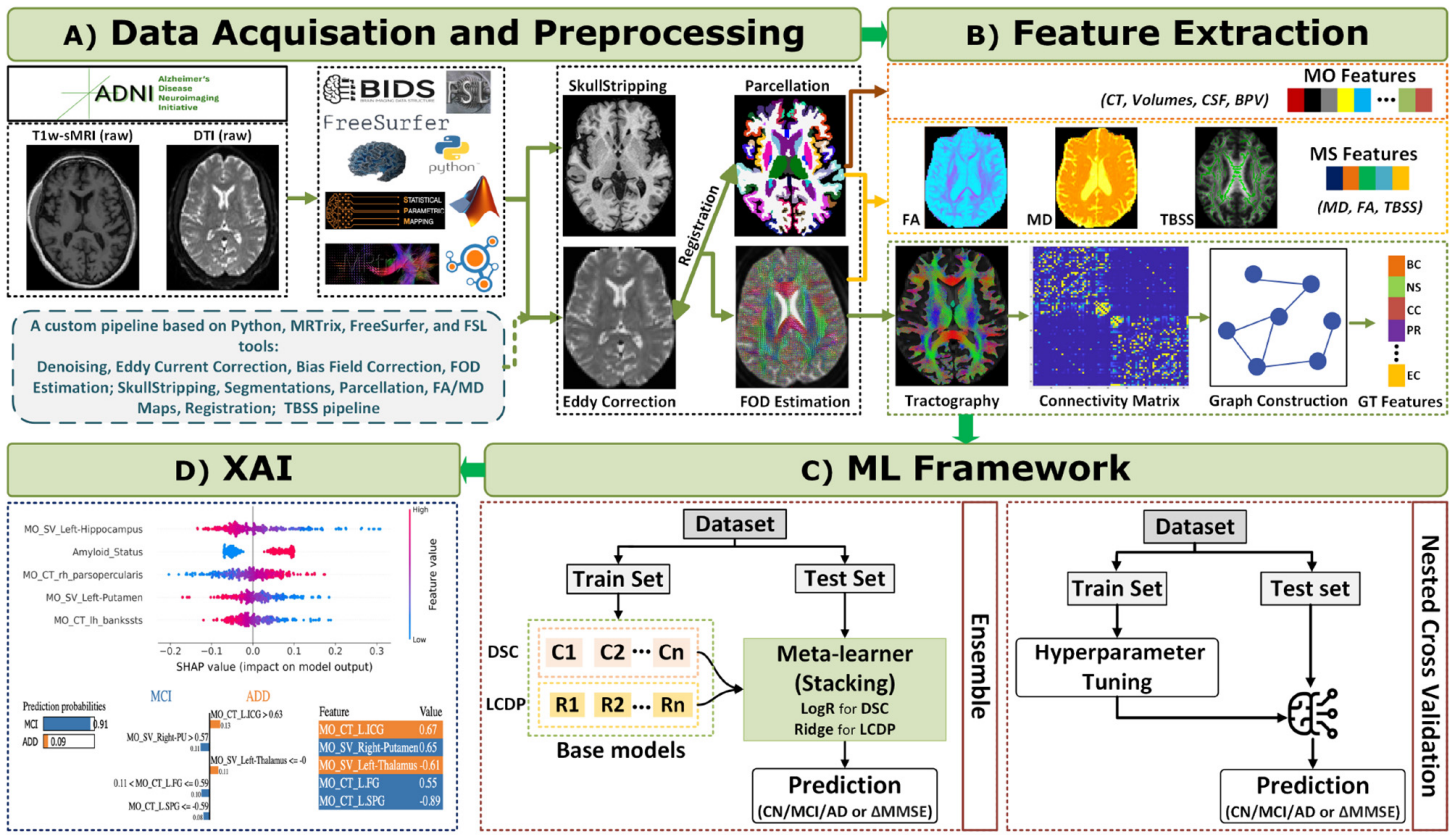
The comprehensive illustration of the ADD diagnosis and cognitive decline prediction framework. Phase 1 includes data preprocessing with T1w-sMRI and dMRI scans for whole-brain tractography and connectivity matrix construction, where dMRI provides connectivity fiber data, and the matrix is derived by discretizing these fibers. Phase 2 involves feature extraction: MO features from T1w-sMRI using FreeSurfer; MS features from T1w-sMRI and dMRI using FreeSurfer, FSL, and MRTrix; and GT features from connectivity matrices by representing Desikan-Killiany (DK) regions as nodes and white matter bundles as edges. The explication of the connectivity matrix as a graph enables the extraction of the GT features. Phase 3 encompasses ML model building with the NestedCV module comprising hyperparameter optimization, DSC (ADD diagnosis) or LCDP (ΔMMSE prediction) tasks, and model performance evaluation; culminating in ensemble models that combine base classifiers via stacking. Phase 4 interprets predictions using XAI tools like SHAP and LIME to enhance model transparency.

Our research methodology followed a systematic and modular ML framework comprising a four-phase approach, as outlined in Figure 1, where each block corresponds to a distinct phase. Phase 1 involved data collection and preprocessing; phase 2 focused on feature extraction; and phase 3 encompassed ML model development, including model training and model performance evaluation using a nested cross-validation (NestedCV) module for DSC tasks and LCDP task. This phase also included the construction of an ensemble module by selecting the best-performing base classifiers or regressors, followed by training and performance evaluation for each classification and prediction task. Phase 4 addressed the explainability and interpretability of ML predictions using XAI approaches, including SHAP and LIME. Details of each phase are provided in the following subsections.

### 2.1 Population description

The dataset used in this study was obtained from the open-source database named ADNI^1^ (Jack Jr et al., 2008). We included imaging data (T1w-sMRI, dMRI) and non-imaging data, including age, gender, clinical, and cognitive scores, along with Amyloid-β status (*Aβ*–/*Aβ*+, to include also subjects outside the ADD continuum). We collected 394 subjects, including 228 CN, 120 MCI patients, and 46 ADD patients, for DSC tasks. The study cohort size distribution and demographic data can be found in Table 1. Furthermore, a subset of 155 subjects, comprising 88 CN, 47 MCI, and 20 ADD, was used for the LCDP task in terms of Mini-Mental-State Examination (MMSE) reduction at follow-ups. To quantify longitudinal cognitive change, we computed the annualized change in MMSE (ΔMMSE) as the difference between an MMSE score obtained at a given visit and a subsequent follow-up MMSE score, normalized by the time elapsed in years between the two visits (Equation 1). On average, the follow-up period was 6.6 years.


$$\begin{array}{l} \Delta MMSE=\frac{MMSE_{follow-up}-MMSE_{visit}}{Months_{elapsed}/12} \end{array}$$ (1)

Where *MMSE*_*visit*_ and *MMSE*_*follow*−*up*_ represent the MMSE scores at a given visit and a subsequent follow-up visit, respectively. The term *Months*_*elapsed*_ represents the number of months elapsed between the two visits.

**Table 1:** Demographic and clinical characteristics of the study population.

**Group (n)**	**Gender (M/F)**	**Age (yrs)**	**Education (yrs)**	**MMSE**	**MoCA**	**ADAS13**	**CDRSB**	**APOE4 (0/1/2)**	***Aβ* status (*Aβ*+/*Aβ*–)**
**(A) Baseline characteristics of the study population used for Alzheimer's disease cognitive stage classification (DSC) tasks**									
CN (228)	129/99	73.48 ± 7.51_[55.10 − 95.40]_	16.71 ± 2.23_[12 − 20]_	28.89 ± 1.35_[23 − 30]_	26.20 ± 2.67_[16 − 30]_	8.27 ± 4.38_[0 − 30.33]_	0.09 ± 0.24_[0 − 1.50]_	164/59/5	138/90
MCI (120)	55/65	74.65 ± 8.57_[55.80 − 93.20]_	16.13 ± 2.65_[8 − 20]_	27.69 ± 2.11_[20 − 30]_	22.87 ± 3_[14 − 29]_	14.69 ± 6.32_[2.33 − 32]_	1.27 ± 1.06_[0 − 5.50]_	82/32/6	60/60
ADD (46)	22/24	75.48 ± 9.30_[55.30 − 95.90]_	15.35 ± 2.51_[12 − 20]_	21.39 ± 4.27_[9 − 28]_	16.17 ± 4.98_[2 − 25]_	30.24 ± 9.27_[13.16 − 54.67]_	5.17 ± 2.58_[1.50 − 14]_	19/22/5	9/37
All (394)	206/188	74.07 ± 8.08_[55.10 − 95.90]_	16.38 ± 2.43_[8 − 20]_	27.65 ± 3.16_[9 − 30]_	24.02 ± 4.48_[2 − 30]_	12.79 ± 9.03_[0 − 54.67]_	1.04 ± 1.92_[0 − 14]_	265/113/16	207/187
**(B) Baseline characteristics of the study population used for longitudinal cognitive decline prediction (LCDP) task**									
CN (88)	45/43	77.24 ± 7.09_[62.70 − 95.40]_	16.49 ± 2.19_[12 − 20]_	28.89 ± 1.39_[23 − 30]_	26.28 ± 2.78_[16 − 30]_	8.36 ± 4.64_[0.33 − 30.33]_	0.12 ± 0.31_[0 − 1.50]_	62/24/2	50/38
MCI (47)	20/27	78.74 ± 8.15_[61.20 − 93.20]_	15.96 ± 2.93_[8 − 20]_	27.60 ± 2.06_[22 − 30]_	23.02 ± 2.81_[17 − 28]_	15.54 ± 6.50_[2.33 − 32]_	1.34 ± 1.26_[0 − 5.50]_	32/13/2	25/22
ADD (20)	10/10	79.67 ± 7.82_[66.20 − 95.90]_	15.70 ± 2.90_[12 − 20]_	20.25 ± 5.78_[9 − 28]_	16.75 ± 6.18_[2 − 25]_	30.38 ± 11.64_[13.16 − 54.67]_	6.17 ± 2.97_[2 − 14]_	11/9/-	3/17
All (155)	75/80	78.01 ± 7.53_[61.20 − 95.90]_	16.23 ± 2.53_[8 − 20]_	27.38 ± 3.80_[9 − 30]_	24.06 ± 4.64_[2 − 30]_	13.38 ± 9.74_[0.33 − 54.67]_	1.27 ± 2.34_[0 − 14]_	105/46/4	78/77

Values are reported as mean ± SD[range] for continuous variables and counts for categorical variables, as indicated. For the LCDP task, only subjects with an available MMSE assessment at a given visit and at least one subsequent follow-up MMSE were included to compute the annualized ΔMMSE.

*Aβ*, Amyloid-β status (*Aβ*+/*Aβ*–); ADD, Alzheimer's disease dementia; ADAS13, Alzheimer's Disease Assessment Scale (13 items); APOE4, ε4 allele of the Apolipoprotein E (APOE) gene; CDRSB, Clinical Dementia Rating–Sum of Boxes; CN, cognitively normal; MCI, mild cognitive impairment; MMSE, Mini-Mental State Examination; MoCA, Montreal Cognitive Assessment; SD, Standard Deviation; M, male; F, female.

### 2.2 Data pre-processing

#### 2.2.1 T1w-sMRI processing

The initial preprocessing step for both neuroimaging modalities—T1w-sMRI and dMRI—involved converting neuroimaging scans from DICOM to NIFTI format. T1w-sMRI scans were processed using FreeSurfer (v7.4.1)^2^ (Fischl, 2012) with its standard recon-all pipeline (Fischl et al., 2002). This pipeline included motion correction, intensity normalization, brain extraction, segmentation, cortical surface reconstruction, and cortical parcellation using the Desikan-Killiany (DK) atlas. The DK atlas defines 84 anatomical regions of interest (ROIs)-comprising 68 cortical and 16 subcortical regions-used for ROI-based dMRI connectivity analysis. For MO feature extraction, additional subcortical/ventricular and corpus callosum measures were obtained from FreeSurfer segmentation outputs (as detailed in Section 2.3.1).

#### 2.2.2 Diffusion MRI processing

The dMRI data were processed using an MRtrix3 software package-based pipeline^3^ (Tournier et al., 2019), adapted from methods previously described (Tahedl, 2024; Oxtoby et al., 2017). Preprocessing included motion correction, eddy-current correction, and bias field correction using tools from FSL (Smith et al., 2004) and ANTs (Avants et al., 2011). Diffusion tensors were then estimated to model water diffusion across white matter. Whole-brain probabilistic tractography was performed using the iFOD2 algorithm with Anatomically Constrained Tractography (ACT) using default parameters (maximum streamline length = 250 mm, minimum cutoff value = 0.06, number of streamlines = 10M) (Tournier et al., 2019; Feinberg, 2024). Streamlines were refined using the SIFT2 (Smith et al., 2015) filtering algorithm to improve biological accuracy and consistency across subjects. Tractography results were registered to the corresponding T1w-sMRI using the anatomical segmentation from FreeSurfer to construct individual structural connectomes. Features such as fiber count, average streamline length, and other microstructural properties were computed to quantify inter-regional connectivity.

#### 2.2.3 Connectivity matrix generation

For each subject, an 82 × 82 structural connectivity matrix was generated using the DK atlas, excluding the left and right cerebellar cortex due to their relative anatomical isolation. Each row and column of the matrix corresponds to a brain region, and each cell represents the connection strength—typically measured by fiber count or related metrics—between a given pair of regions. A complete list of the 84 DK atlas brain regions (including the 82 used in this study) is provided in Supplementary Table S1.

### 2.3 Feature extraction

We extracted MO and MS features to describe the individual topology of brain networks and GT metrics from brain networks.

#### 2.3.1 Morphometric (MO) features

MO features were extracted from T1w-sMRI scans processed with the FreeSurfer pipeline, as described in Section 2.2. This included volumetric measures for subcortical regions and cortical thickness measures for selected cortical areas (Fischl et al., 2002).

- Subcortical volumes (SV): the volumetric features included gray-matter (GM) structures from both hemispheres (thalamus, caudate, putamen, pallidum, hippocampus, amygdala, and nucleus accumbens), ventricular volumes, and corpus callosum subdivisions, yielding 22 SV features.
- Cortical thickness (CT): CT measurements were obtained for 68 cortical brain regions (34 per hemisphere) (Busovaca et al., 2016; Dickerson et al., 2009).
- Other measures: Brain Parenchymal Volume (BPV) volume was also included as an additional MO feature. We computed BPV using FSL's FAST tool (Zhang et al., 2001), which segments T1w-sMRI scans into GM, white matter (WM), and CSF. BPV was defined as the sum of GM and WM volumes.

In total, 91 MO features were extracted per subject for downstream analysis.

#### 2.3.2 Microstructural (MS) features

MS brain features were extracted from preprocessed T1w-sMRI and dMRI scans. These features included:

- Whole-brain mean diffusivity (MD) and fractional anisotropy (FA): a brain mask was first generated from the dMRI scans using FSL's BET tool (Smith, 2002). The diffusion tensor model was then fitted using DTIFIT, producing FA and MD maps. These maps were used in conjunction with the brain mask to calculate whole-brain mean FA and mean MD.
- Hippocampal Mean MD (left and right): Left and right hippocampal segmentations from T1w-sMRI (via FSL's FIRST Patenaude et al., 2011) were registered to Diffusion Tensor Imaging (DTI) space using FLIRT. These masks were overlaid on the MD map (computed using MRtrix's dwi2tensor and tensor2metric) to extract hippocampal MD values. Summary statistics were calculated using the mrstats command, and we retained the mean MD for both left and right hippocampi.
- Mean FA within the Tract-Based Spatial Statistics (TBSS) white matter skeleton: The mean FA within the TBSS-derived white matter skeleton was calculated using the standard TBSS pipeline in FSL (Smith, 2002).

In total, 5 MS features were extracted per subject.

#### 2.3.3 Graph-theoretical (GT) features

A graph is defined as G = (V, E), where V represents a set of vertices (or nodes), and E denotes a set of edges connecting these nodes (Zhang et al., 2024). In this study, subject-specific brain graphs were constructed using 82 brain regions from the DK atlas (see Section 2.2), where each region was treated as a node. Edges represented WM fiber bundles between regions, as derived from whole-brain tractography. Although the number of nodes (82) remained constant across subjects—due to consistent atlas-based parcellation—the number and strength of edges varied, reflecting inter-subject differences in structural connectivity. Edges were weighted by the corresponding connection strength derived from tractography, resulting in weighted undirected graphs. The resulting connectivity matrix for each subject encoded pairwise connection strengths, forming the basis for computing GT metrics. These subject-specific graphs formed the basis for extracting GT features that characterize brain network topology.

For each subject-specific graph, both local and global GT metrics were computed following Rubinov and Sporns (2010) using the NetworkX Python library (Hagberg et al., 2008). For shortest-path–based metrics, edge weights were transformed into distances using their inverse. This resulted in a combined GT feature vector of 591 features per subject. A complete list of GT features and abbreviations is provided in Supplementary Table S2.

- Nodal (local) GT Metrics: these metrics were computed for each of the 82 brain regions (nodes) based on the DK atlas. Seven node-wise features were extracted: clustering coefficient (CC), degree centrality (DC), eigenvector centrality (EC), closeness centrality (CClo), betweenness centrality (BC), node strength (NS), and PageRank (PR), resulting in 82 × 7 = 574 local features per subject.
- Global GT Metrics: these were computed across the entire brain network and included 17 global measures: density, modularity, assortativity, transitivity, global efficiency, characteristic path length (CPL), diameter, small-worldness (SW), degree distribution entropy (DegEnt), spectral radius (SpecRad), average degree (avgDeg), average clustering coefficient (avgCC), average betweenness centrality (avgBC), average eigenvector centrality (avgEC), average closeness centrality (avgCClo), average node strength (avgNS), and average PageRank (avgPR).

In addition, the Amyloid-β status (*Aβ*–/*Aβ*+) was incorporated as a feature in all experiments across both DSC and LCDP pipelines. It was consistently integrated with each feature subset (MO, MS, GT) to ensure model comparability and capture amyloid-related pathophysiological patterns. In total, the feature set consisted of 91 MO features, 5 MS features, 591 GT features, and 1 *Aβ* status. This yielded a final input feature vector of 688 elements per subject.

### 2.4 Feature pre-processing and covariate adjustment

The MO, MS, and GT features were preprocessed to control for nuisance covariates while preserving the integrity of the NestedCV framework. Initially, in order to reduce anatomical variability, the volumetric MO features (i.e., subcortical volume) were corrected for head size by normalizing each regional volume by the estimated total intracranial volume (eTIV), as Equation 2:


$$\begin{array}{l} {\tilde{x}}_{i,j}=\frac{x_{i,j}}{{\text{eTIV}}_{i}} \end{array}$$ (2)

where *x*_*i, j*_ denotes the raw subcortical volume of the region *j* for subject *i*, and ${\tilde{x}}_{i,j}$ is the eTIV-normalized value. However, cortical MO features (i.e., cortical thickness) were not eTIV-normalized (Buckner et al., 2004; Whitwell et al., 2001).

After eTIV correction, all imaging-derived features (MO, MS, and GT) were residualized with respect to age and sex–to prevent confounding effects in predictive modeling–within each training fold only (Snoek et al., 2019; Varma and Simon, 2006). Specifically, for each imaging feature *k*, a linear regression model was fit on the training data, as in Equation 3:


$$\begin{array}{l} x_{i,k}=\beta_{0,k}+\beta_{1,k}{\text{Age}}_{i}+\beta_{2,k}{\text{Sex}}_{i}+\varepsilon_{i,k} \end{array}$$ (3)

and residualized feature values were computed as in Equation 4:


$$\begin{array}{l} {\hat{x}}_{i,k}=x_{i,k}-\left(\beta_{0,k}+\beta_{1,k}{\text{Age}}_{i}+\beta_{2,k}{\text{Sex}}_{i}\right) \end{array}$$ (4)

The regression parameters $\left({\hat{\beta }}_{0},{\hat{\beta }}_{1},{\hat{\beta }}_{2}\right)$ estimated from the training fold were subsequently applied to the corresponding held-out test fold to avoid data leakage. Following residualization, all features were standardized in a fold-wise manner prior to model training. For each feature *k*, standardization was performed using the mean and standard deviation estimated from the training data, as in Equation 5:


$$\begin{array}{l} z_{i,k}=\frac{{\hat{x}}_{i,k}-\mu_{k}^{\text{train}}}{\sigma_{k}^{\text{train}}} \end{array}$$ (5)

where ${\hat{x}}_{i,k}$ denotes the residualized feature value for subject *i*, and $\mu_{k}^{\text{train}}$ and $\sigma_{k}^{\text{train}}$ are the mean and standard deviation of feature *k* computed from the training fold. The same parameters were applied to the corresponding test fold. This feature preprocessing ensures control of head size, age, and sex effects, allowing subsequent ML models to learn disease-related variation rather than nuisance covariation with age or sex.

### 2.5 Proposed ML framework

#### 2.5.1 Base learners and nested cross-validation

We presented a systematic ML framework to investigate the discriminative power of diverse feature subsets for Alzheimer's DSC and LCDP tasks. The main aim of the study was to examine how the stepwise inclusion of feature subsets, starting with MO features and subsequently adding MS and GT features, impacts predictive accuracy and interpretability. The framework is designed to evaluate the predictive performance of various ML models across these feature subsets and to identify the most informative features for each task. For DSC, we employed seven heterogeneous classifiers to ensure robust generalization and to capture a wide spectrum of learning biases. These included linear models such as logistic regression (LogR) and linear discriminant analysis (LDA); tree-based models including decision tree (DT), random forest (RF), adaptive boosting (AdaBoost), and extreme gradient boosting (XGB); and the kernel-based model support vector machine (SVM). For LCDP, we used seven regression models: Ridge, DT, RF, AdaBoost, XGB, Gaussian Process Regressor (GPR), and Support Vector Regressor (SVR).

**Figure 2:**
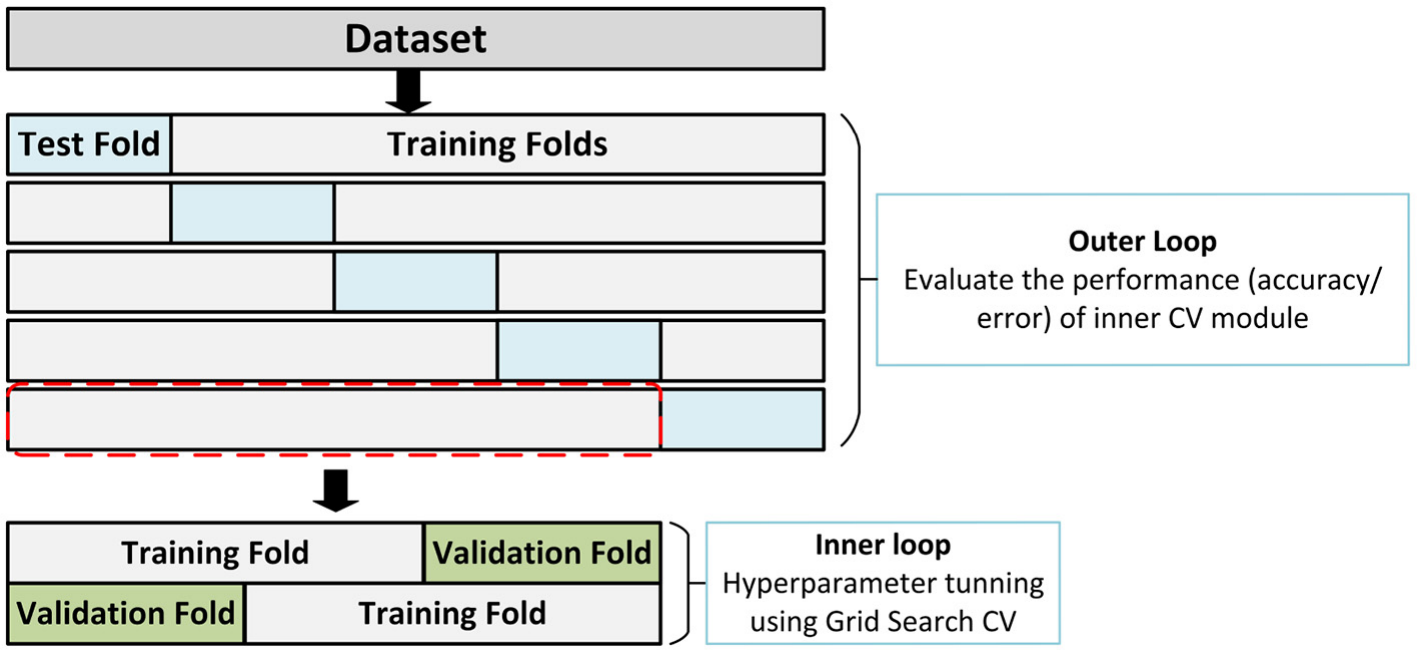
Schematic representation of the NestedCV module. The inner CV loop performs hyperparameter tuning using grid search on a multi-dimensional parameter grid and optimizes hyperparameters for each outer fold. The outer CV ensures the unbiased performance evaluation of the inner CV.

In ML, hyperparameter optimization encompasses the challenge of identifying the optimal combination of parameters for a model, which is an essential phase in the model selection process. The aim is to identify the optimal hyperparameter configuration for each ML model by optimizing a performance metric, such as accuracy or error (Elgeldawi et al., 2021; Probst et al., 2019). In this study, we used a grid search strategy to optimize hyperparameters. The grid search strategy systematically explores a grid of parameters produced from a predefined subset of an ML model's hyperparameter space, intending to identify the combination of hyperparameters that optimizes the model's predictive performance. The complete hyperparameter grids explored for each model are reported in Supplementary Table S3. We incorporated a NestedCV into the training pipeline for model selection and performance estimation, with the grid search created as part of the 5-fold NestedCV module. Consequently, the hyperparameter optimization was performed through grid search during a 3-fold inner CV, and the optimal set of parameters for each model was identified. Additionally, an external 5-fold NestedCV was employed to ensure the unbiased performance evaluation of the inner CV for the DSC and LCDP tasks. In the 5-fold NestedCV, the model was trained over five iterations for each task, with each iteration involving three phases: (i) hyperparameter optimization, (ii) model training, and (iii) predictions and performance evaluation. Training was conducted on four data folds (4 = 5–1), with the fifth fold reserved for evaluation, ensuring robust model assessment (Figure 2). The NestedCV module's outer CV further evaluated the accuracy and performance of the inner CV process, providing an unbiased estimate of the ML model's true error and performance (Bates et al., 2024).

#### 2.5.2 Ensemble learning

Following NestedCV, the ensemble learning framework was constructed for each task by combining base learners using a performance- and diversity-aware strategy. This framework was designed to enhance predictive robustness and reduce fold-wise variability by integrating complementary model behavior. Ensembles were built separately for each DSC and LCDP task and feature set using base models trained under identical NestedCV splits, ensuring consistent alignment of subject-level out-of-fold (OOF) predictions.

For a given task *t* and feature set *f*; $M_{t,f}=\left\{M_{1},\ldots ,M_{K}\right\}$ denote the pool of base models obtained from NestedCV. Model diversity was assessed by computing pairwise Pearson correlations between OOF predictions, defined for two models *M*_*m*_ and *M*_*n*_ as:


$$\begin{array}{l} \rho_{\text{pred}}\left(m,n\right)=\text{corr}\left({\text{p}}^{\left(m\right)},{\text{p}}^{\left(n\right)}\right) \end{array}$$ (6)

where **p**^(*m*)^ represents the vector of predicted class probabilities (DSC) or continuous outcomes (LCDP). Models exhibiting high redundancy were pruned using a correlation threshold (τ = 0.85), whereby among highly correlated pairs, the model with inferior cross-validated performance was discarded. This procedure yielded a reduced, diversity-preserving subset $M_{t,f}^{*}$.

Selected base models were combined using stacked generalization. For each subject *i*, a meta-feature vector was defined as:


$$z_{i}=\{\begin{array}{ll} \left[p_{i}^{\left(1\right)},p_{i}^{\left(2\right)},\ldots ,p_{i}^{\left(|M^{*}|\right)}\right], & \text{DSC}\\ \left[{\hat{y}}_{i}^{\left(1\right)},{\hat{y}}_{i}^{\left(2\right)},\ldots ,{\hat{y}}_{i}^{\left(|M^{*}|\right)}\right] & \text{LCDP} \end{array}$$ (7)

For DSC, a logistic regression meta-classifier was trained, whereas for LCDP, a ridge regression meta-model was employed. Meta-models were trained exclusively on pooled OOF predictions to prevent data leakage.

To preserve interpretability, ensemble-level feature importance was computed by propagating base-model coefficients through the stacking weights. For each original feature *j*, importance was defined as:


$$\begin{array}{l} I_{j}=\underset{m\in M^{*}}{\sum }|w_{m}|\cdot |\beta_{j}^{\left(m\right)}| \end{array}$$ (8)

where *w*_*m*_ denotes the meta-model coefficient associated with the base model *M*_*m*_ and $\beta_{j}^{\left(m\right)}$ the corresponding base-model feature coefficient. Importance scores were normalized and ranked. Ensemble performance was evaluated using the same fold-wise metrics as individual models.

#### 2.5.3 Statistical analysis

We conducted paired statistical analyses using fold-wise cross-validation results obtained from ensemble models in order to assess whether incremental inclusion of multimodal MRI feature sets resulted in statistically meaningful performance improvements. For each task, statistical significance was tested using paired, one-sided Wilcoxon signed-rank tests across cross-validation folds (Wilcoxon, 1945; Dietterich, 1998). Specifically, we tested whether model performance improved when adding MO with MS features (MO vs. MO+MS), and the GT feature set to MO+MS (MO+MS vs. MO+MS+GT). These comparisons were defined a priori and were hypothesis-driven, reflecting the focus of this systematic study. For each paired comparison, the Wilcoxon signed-rank statistic *W* was computed as Equation 9:


$$\begin{array}{l} W=\underset{i:d_{i}>0}{\sum }\mathrm{rank}\left(\left|d_{i}\right|\right) \end{array}$$ (9)

Where *d*_*i*_ = *x*_*i*_−*y*_*i*_, denotes the fold-wise difference in performance between two models for fold *i*, with *x*_*i*_ and *y*_*i*_ representing the evaluation metric obtained using different feature sets. For DSC tasks, statistical testing was performed using BACC; whereas for LCDP, regression performance was assessed using the MAE. Statistical significance was defined at α at a level of 0.05. In addition to hypothesis testing, we employed bootstrapped resampling (1,000 iterations) to estimate 95% confidence intervals (CI) for all evaluation metrics. Bootstrapping was applied to OOF predictions and used to quantify the robustness and uncertainty of model performance estimates, including the generation of confidence bands for ROC curves.

#### 2.5.4 eXplainable artificial intelligence

To improve the transparency and interpretability of ML model predictions, explainable artificial intelligence (XAI) methods were integrated into the proposed framework. In addition to conventional performance evaluation metrics, we evaluated the explainability and interpretability of model predictions. Although ML models can achieve high predictive performance, their complex decision-making processes (often referred to as “black boxes”) can limit interpretability, particularly in high-dimensional neuroimaging applications (Hassija et al., 2024). To enhance transparency and user trust, XAI methods were employed to identify and quantify the contribution of individual MRI-derived features to model predictions across each DSC and LCDP task.

Specifically, we employed SHapley Additive exPlanations (SHAP) and Local Interpretable Model-Agnostic Explanations (LIME), which provide complementary perspectives on model behavior. SHAP was used to derive global explanations by estimating the average contribution of each feature to model outputs based on cooperative game theory, thereby enabling identification of the most influential predictors across the entire dataset (Salih et al., 2025). In contrast, LIME was applied to generate local explanations by highlighting how each feature contributes to an individual model prediction, which allows inspection of feature contributions at the subject level (Ribeiro et al., 2016). In addition to SHAP and LIME, we also examined feature importance using ensemble-based model importance measures for both DSC and LCDP tasks. These model-derived importance rankings provide complementary, task-specific insights and help mitigate reliance on any single explainability method. In addition to SHAP and LIME, feature relevance was also assessed using model-based importance measures derived from the ensemble learners for both DSC and LCDP tasks, providing complementary, task-specific insights into influential features. Together, these XAI analyses enabled a systematic and interpretable evaluation of how different feature sets (i.e., MO, MS, and GT) contributed to model performance. This approach provided biologically and clinically meaningful insights into feature relevance while complementing conventional performance-based evaluation.

### 2.6 Experimental setup

Initially, we evaluated the performance of each ML model using all features from each category separately—MO, MS, and GT. Following this, we constructed nested feature sets: MO+MS, MO+GT, MS+GT, and MO+MS+GT to systematically examine the impact of increasing feature complexity. This modular approach allowed for a comprehensive comparison of model performance and feature importance as more feature subsets were added. Overall, this exhaustive ablation analysis resulted in 28 distinct classification and regression tasks, enabling the identification of the best-performing classifiers for the DSC tasks and regression models for the LCDP task, along with the most optimal set of features. An 80/20 train-test split was employed, where 80% of the data was used for training the models. Hyperparameter optimization and model selection were performed on the training set using 5-fold NestedCV combined with GridSearchCV. The remaining 20% of the data was held out as an independent hold-out test set to evaluate the final performance of the trained models, ensuring strict separation between training and testing phases.

The predictive performance of base estimators and ensemble learners was evaluated using a variety of diverse metrics. In the DSC pipeline, the base classifiers and ensemble learner were evaluated using accuracy (ACC), balanced accuracy (BACC), sensitivity (SEN), specificity (SPE), and Receiver Operating Characteristic—Area Under the Curve (rocAUC). These metrics provided a detailed understanding of each classifier's overall accuracy, as well as its ability to correctly identify both positive and negative cases. For the LCDP pipeline, the base regression models and ensemble learners were evaluated using Mean Absolute Error (MAE), Mean Squared Error (MSE), Root Mean Squared Error (RMSE), and the Coefficient of Determination (R^2^) as evaluation metrics.

## 3 Experiments and results

### 3.1 Alzheimer's disease cognitive stage classification tasks

For the CN vs. ADD classification task, the dataset included 274 subjects (228 CN, 46 ADD), split into 219 for training and 55 for evaluation. The CN vs. MCI task had 348 subjects (228 CN, 120 MCI) with a 278/70 split, while the MCI vs. ADD task included 166 subjects (120 MCI, 46 ADD), split as 133/33. Overall, the results demonstrated that the ensemble learning consistently improves classification robustness and outperforms all individual base classifiers, particularly in CN vs. ADD and MCI vs. ADD tasks. Table 2 summarizes the best-performing model–feature set combinations for each DSC task, reporting BACC (as the primary metric), ACC, and rocAUC as mean ± standard deviation based on NestedCV. The complete performance results for all models, feature sets, and evaluation metrics are provided in the Supplementary Material (Supplementary Tables S4A–C). Furthermore, the receiver operating characteristic (ROC) curves comparing classification performance across MRI-derived feature sets for the DSC task are provided in the Supplementary Material (Supplementary Figures S1A–C).

**Table 2:** Summary of best-performing models and feature sets for disease stage classification (DSC) and longitudinal cognitive decline prediction (LCDP) tasks.

**Task**	**Feature set**	**Model**	**BACC/MSE**	**ACC/RMSE**	**rocAUC/R^2^**
CN–ADD	MO+MS	Ensemble	0.898 ± 0.051	0.920 ± 0.030	0.959 ± 0.029
MCI–ADD	MO	Ensemble	0.769 ± 0.116	0.771 ± 0.097	0.832 ± 0.085
CN–MCI	MO	SVM	0.652 ± 0.044	0.704 ± 0.029	0.656 ± 0.093
LCDP	MO	Ensemble	0.531 ± 0.307	0.701 ± 0.221	0.212 ± 0.177

Values are reported as mean ± standard deviation across cross-validation folds. For DSC tasks, balanced accuracy (BACC), accuracy (ACC), and rocAUC are reported, where BACC is considered the primary metric.

For LCDP, performance is summarized using the mean squared error (MSE), root mean squared error (RMSE), and coefficient of determination (*R*^2^), where MSE is considered the primary metric.

For each task, the model and feature set shown correspond to the highest cross-validated performance. Complete performance results for all models and feature sets are provided in the Supplementary Tables S4A–D). DSC, disease stage classification; LCDP, longitudinal cognitive decline prediction; CN, cognitively normal; MCI, mild cognitive impairment; ADD, Alzheimer's disease dementia; MO, morphometric features; MS, microstructural features; SVM, support vector machine.

For the CN vs. ADD classification, the ensemble model achieved the highest classification accuracy of 0.942 ± 0.024 (BACC: 0.868 ± 0.052; rocAUC: 0.951 ± 0.034) using the MO feature set alone. The classification performance declined when using the ensemble learner with the MS feature set alone, which yielded a BACC of 0.774 ± 0.025. In contrast, the ensemble learner with the GT feature set alone yielded a comparatively improved BACC (0.820 ± 0.097). In comparison, the ensemble learner using a combination of MO and MS feature set achieved the highest overall performance with BACC of 0.898 ± 0.051 (SEN: 0.867 ± 0.093; rocAUC: 0.959 ± 0.029). Likewise, the ensemble learner using MO+GT feature sets yielded a strong performance (BACC: 0.887 ± 0.060). However, incorporating additional features such as the combination of MO, MS, and GT feature sets did not yield further improvement, and an ensemble learner yielded a BACC (0.866 ± 0.057), which is slightly lower compared to MO+MS. These findings highlighted that this multimodal integration slightly outperformed the unimodal approaches. Specifically, these results suggested that incorporating MO with the MS feature set contributes slightly to distinguishing between cognitively normal individuals and those with ADD.

For the MCI vs. ADD classification, the ensemble learners consistently achieved the highest classification performance (i.e., BACC, rocAUC) across all feature sets. The MO feature set achieved superior results compared to the MS and GT features alone. The ensemble learner yielded the highest BACC of 0.769 ± 0.116 (ACC: 0.771 ± 0.097; SEN: 0.762 ± 0.179; rocAUC: 0.832 ± 0.085). In contrast, the lowest performance was observed using MS features alone, with a BACC of 0.661 ± 0.055. The integration of additional features with the MO feature set (i.e., MO+MS) yielded modest improvement, particularly in terms of classification accuracy (0.795 ± 0.058); however, no combination of feature sets outperformed MO alone.

Likewise, the highest performance for the CN vs. MCI classification task was observed using the MO feature set with SVM, achieving a BACC of 0.652 ± 0.044 (ACC: 0.704 ± 0.029). However, the ensemble learner using MO+MS feature sets provided achieved the highest rocAUC of 0.703 ± 0.103. The ensemble learner using the GT feature set alone again yielded the lowest predictive performance with a BACC of 0.567 ± 0.042. Furthermore, these findings indicate that combining MO features with MS and GT metrics led to a decline in predictive performance, indicating possible redundancy or noise from added features.

In summary, the results indicated that the ensemble learning approach consistently demonstrated improved results across all DSC tasks. This study evaluated the discriminative ability of individual and combined feature sets in all DSC tasks. Furthermore, statistical analysis indicated that incremental increases in feature complexity did not result in statistically significant performance improvements across any of the DSC tasks (Table 3). Although modest numerical trends were observed for certain comparisons (e.g., MO+MS relative to MO in CN–ADD classification), none of the pairwise differences reached statistical significance at α = 0.05. The findings of the study reported that MO features alone demonstrated strong standalone performance, particularly in the MCI vs. ADD and CN vs. MCI classification tasks, where they outperformed all other unimodal and multimodal combinations. In contrast, the integration of multimodal features (MO+MS) provided slight improvements in the CN vs. ADD classification task. These findings highlight the usefulness of the MO features across all DSC tasks.

**Table 3:** Statistical analysis of ensemble model performance across feature sets.

**Task**	**Comparison**	***W*-statistic**	***p*-value**
CN–ADD	MO vs. MO+MS	3	0.906
	MO+MS vs. MO+MS+GT	9	0.072
MCI–ADD	MO vs. MO+MS	7.5	0.179
	MO+MS vs. MO+MS+GT	3	0.767
CN–MCI	MO vs. MO+MS	4	0.844
	MO+MS vs. MO+MS+GT	10	0.313
LCDP	MO vs. MO+MS	6	0.406
	MO+MS vs. MO+MS+GT	3	0.156

Paired, one-sided Wilcoxon signed-rank tests were performed using fold-wise cross-validation results from ensemble models to assess whether performance improved with incremental feature inclusion.

For DSC tasks, BACC was evaluated, while MAE was used for the LCDP task.

Statistical significance was defined at α = 0.05.

### 3.2 Longitudinal cognitive decline prediction task

For the LCDP (ΔMMSE) task, a total of 155 subjects were included (88 CN, 47 MCI, and 20 ADD), split into 124 for training and 31 for evaluation. Among all models assessed in the ablation study, the ensemble model using the MO feature set achieved the best overall performance, with the lowest MSE (0.531 ± 0.307) and RMSE (0.701 ± 0.221), as well as the highest R^2^ (0.212 ± 0.177). However, the lowest MAE (0.396 ± 0.072) was achieved by the AdaBoost using the same feature set (Table 2). The detailed results for the LCDP task across all models and feature sets are presented in Supplementary Table S4D. The results indicated that the ensemble model yielded the lowest MSE and RMSE and the highest R^2^ values for individual feature sets, especially for MO, while AdaBoost yielded a slightly lower MAE. Similar results are observed for the MO+MS and MO+GT feature sets, where the ensemble model yielded lower RMSE (0.704 ± 0.230 and 0.725 ± 0.2) and higher R^2^ values of 0.207 ± 0.178 and 0.141 ± 0.203, respectively. In contrast, the MS and GT feature sets alone yielded a lower predictive performance across all models.

Among the base learners, the DT models consistently showed the most variability and the largest errors across all feature sets. In contrast, AdaBoost and SVR performed competitively, particularly in terms of MAE. In summary, the MO feature set integrated with the ensemble learner yielded the best overall performance. These results suggest that increasing feature complexity did not lead to a statistically significant improvement in predictive performance (Table 3).

### 3.3 Explainability and interpretability

**Figure 3:**
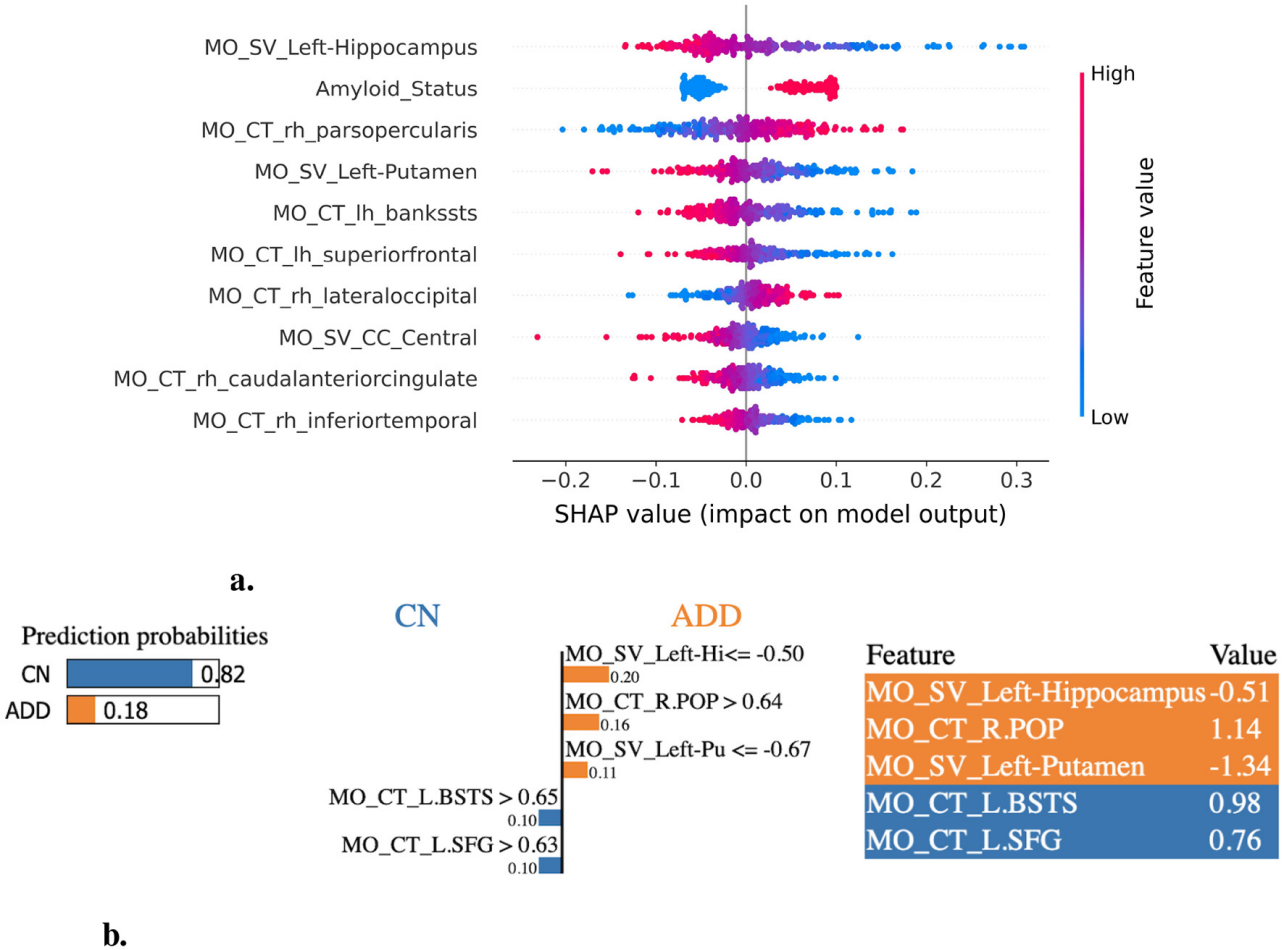
XAI-based feature importance analysis for CN vs. ADD classification task. **(a)** SHAP summary plot illustrating the most influential features contributing to the classification of ADD. **(b)** LIME explanation highlighting key features distinguishing CN individuals from those with ADD. L.HI, Left-Hippocampus; R.POP, ctx-rh-parsopercularis; L.PU, Left-Putamen; L.BSTS, ctx-lh-bankssts; L.SFG, ctx-lh-superiorfrontal.

**Figure 4:**
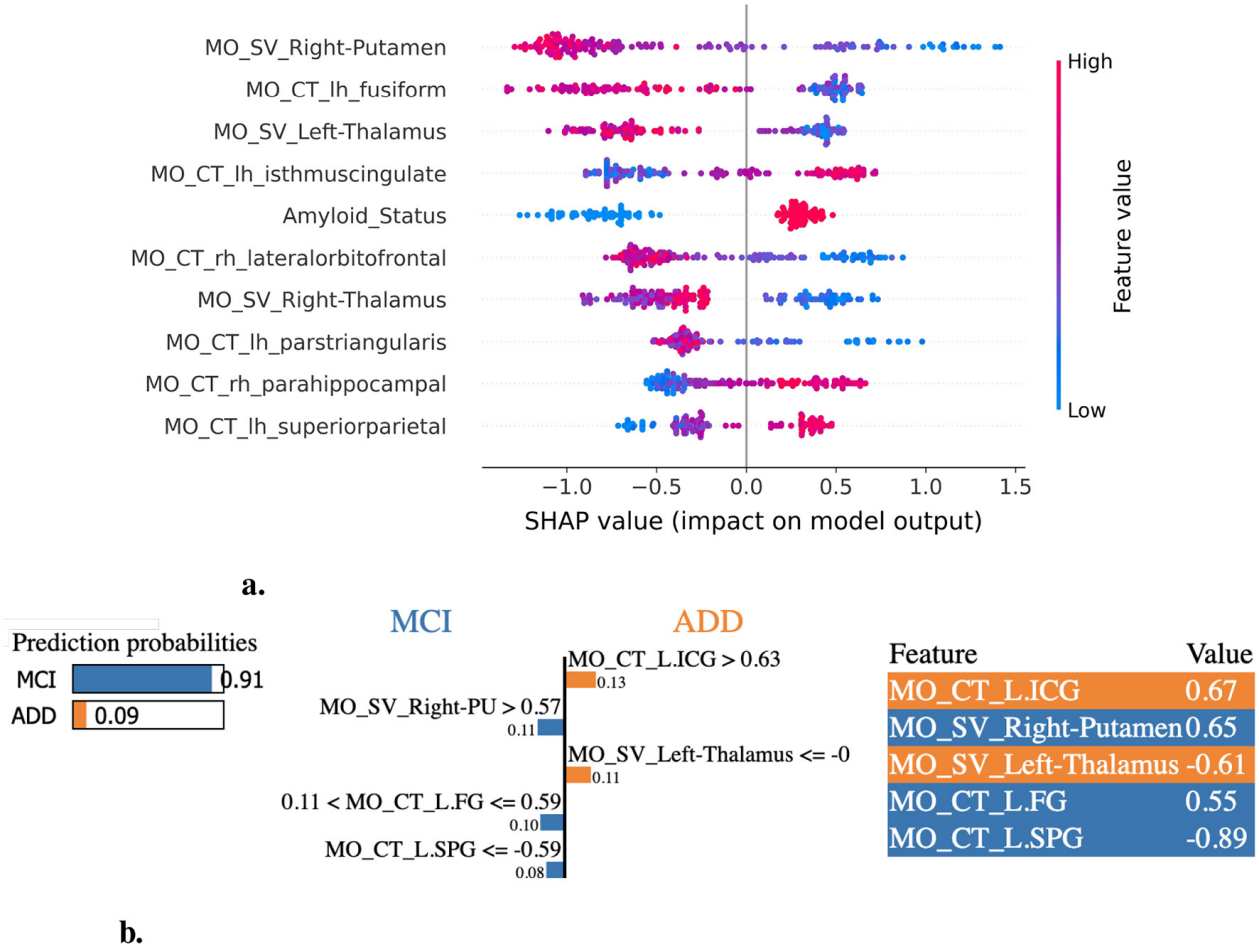
XAI-based feature importance analysis for the MCI vs. ADD classification task. **(a)** SHAP summary plot illustrating the most influential features contributing to the classification of ADD. **(b)** LIME explanation highlighting key features distinguishing MCI individuals from those with ADD. L.ICG, ctx-lh-isthmuscingulate; R.PU, Right-Putamen; L.FG, ctx-lh-fusiform; L.SPG, ctx-lh-superiorparietal.

**Figure 5:**
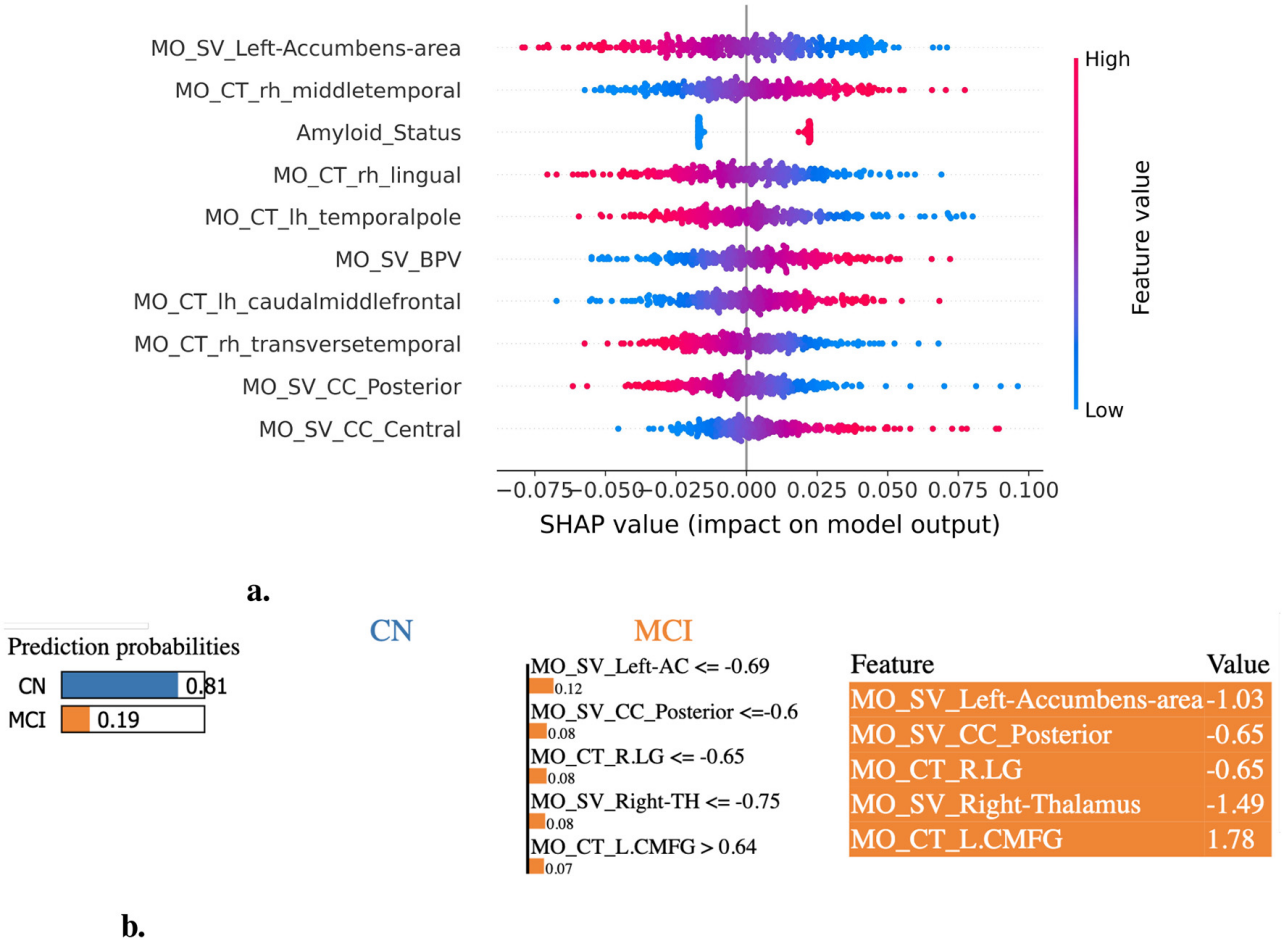
XAI-based feature importance analysis for the CN vs. MCI classification task. **(a)** SHAP summary plot illustrating the most influential features contributing to the classification of MCI. **(b)** LIME explanation highlighting key features distinguishing CN individuals from those with MCI. L.AC, Left-Accumbens-area; R.LG, ctx-rh-lingual; R.TH, Right-Thalamus; L.CMFG, ctx-lh-caudalmiddlefrontal.

We employed XAI methods to assess feature importance in the ML model prediction process. Specifically, we extracted the top 20 most significant features for each DSC and LCDP task, enabling interpretable insights into the key discriminative attributes driving the models. The SHAP and LIME plots highlight the most influential features across the CN vs. ADD (Figure 3), MCI vs. ADD (Figure 4), and CN vs. MCI (Figure 5) classification tasks. The complete list of the top 20 most discriminative features identified by XAI methods and ensemble-based importance measures for each task are listed in Supplementary Tables S5A–D. The amyloid status consistently appeared as a discriminant feature across DSC and LCDP tasks. For the CN vs. ADD task, the most discriminative features identified by SHAP are illustrated in Figure 3a and LIME (Figure 3b). The most consistently identified significant features across feature importance methods include mean diffusivity (MD), mean FA within the TBSS white matter skeleton from the MS feature set, and left hippocampus volume, amyloid status, left putamen volume, and cortical thickness in the inferior temporal and cingulate area from the MO feature set. In addition, amygdala and accumbens volumes, and cortical thickness in the superior frontal and temporal regions were identified by two or more methods (Figures 3a, b, Supplementary Table S5A).

For the MCI vs. ADD task, the most discriminative features identified across feature importance methods include the left hippocampus, right putamen, amygdala (left+right), BPV, and thalamus (left+right) volume, amyloid status, and the cortical thickness in the parahippocampal and entorhinal regions (Figures 4a, b, Supplementary Table S5B). Likewise, the most relevant features for the CN vs. MCI task include the left accumbens, hippocampus (left+right), thalamus (left+right) volume, amyloid status, and the thicknesses of the middle temporal, lingual, precuneus, supramarginal, and fusiform regions (Figures 5a, b, Supplementary Table S5C).

**Figure 6:**
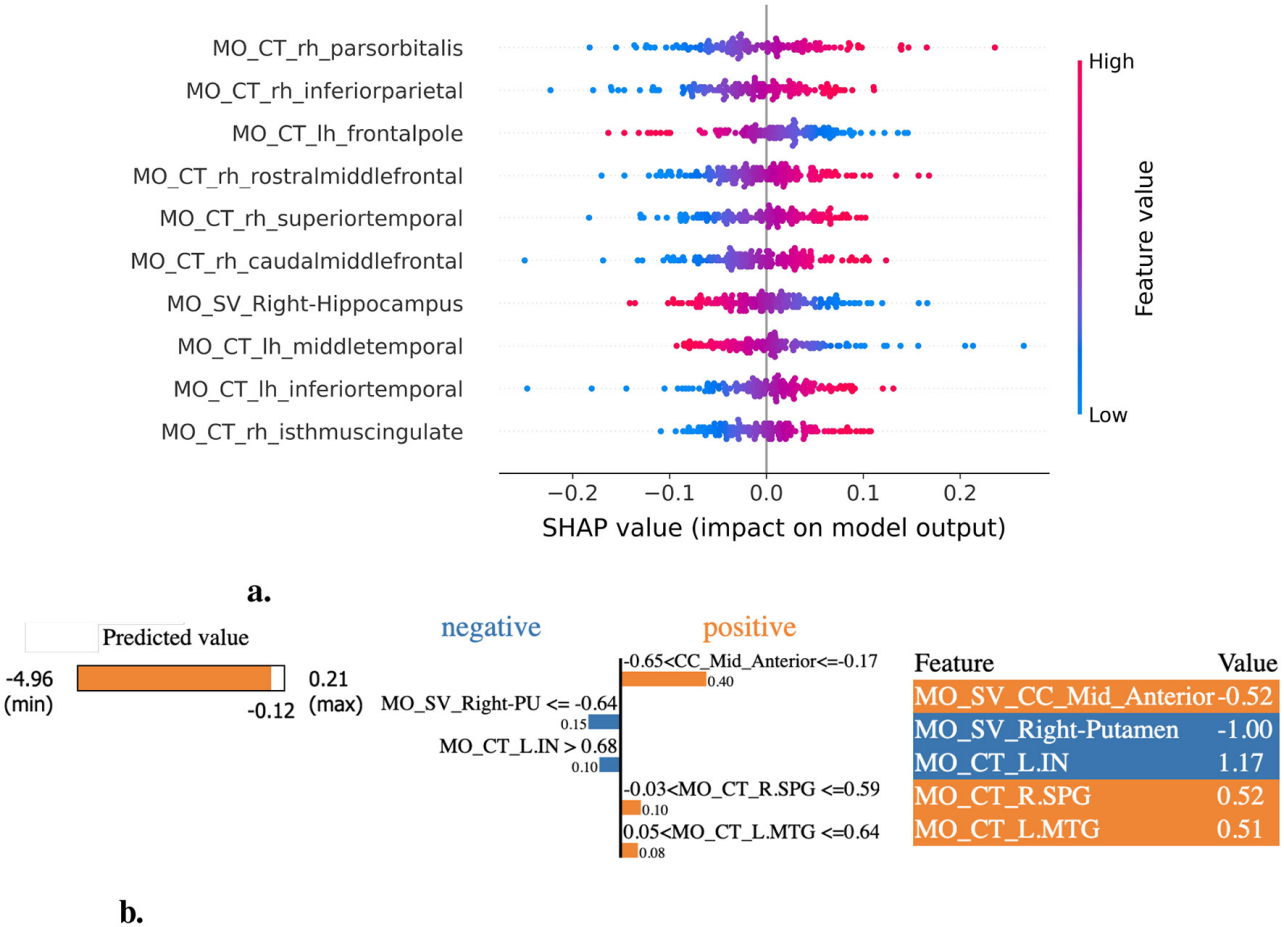
XAI-based feature importance analysis for the LCDP (ΔMMSE) task. **(a)** SHAP summary plot illustrating the most influential features contributing to the LCDP task. **(b)** LIME explanation highlighting key features for the LCDP task. R.PU, Right Putamen; L.IN, ctx-lh-insula; R.SPG, ctx-rh-superiorparietal; L.MTG, ctx-lh-middletemporal.

In the LCDP (ΔMMSE) task, the most important features identified across feature importance methods include cortical thickness within the middle temporal, entorhinal, isthmus cingulate, bankssts, and inferior parietal regions; amyloid status; and subcortical volumes of the putamen (left+right), hippocampus, and right amygdala regions (Figures 6a, b, Supplementary Table S5D).

## 4 Discussion

### 4.1 Interpretation of key findings

This section interprets the main findings of this study and presents a comparative summary (Table 4) of related MRI-based ML studies on Alzheimer's disease. In this study, we introduced an ML-based ensemble learning framework to integrate MRI-derived MO, MS, and GT features for DSC and LCDP tasks. Ensemble models outperformed base classifiers in DSC (in particular, CN vs. ADD and MCI vs. ADD) and LCDP tasks. In contrast, for the CN vs. MCI DSC task, SVM and RF yielded slightly better performance compared to ensemble learners, particularly in terms of BACC.

**Table 4:** Comparative analysis of related MRI-based ML studies on Alzheimer's disease.

**References**	**Dataset (N)**	**Modality**	Task (ACC/BACC^*^)	**Technical details**
Dyrba et al. (2013)	EDSD (280)	dMRI	CN–AD (0.75–0.89)	SVM using dMRI-derived FA and MD features
Ebadi et al. (2017)	ADNI (45)	dMRI	CN–AD (0.80); MCI–AD (0.83); CN–MCI (0.70)	Ensemble using structural connectivity GT metrics
Diogo et al. (2022)	ADNI (570), OASIS (531)	sMRI	CN–AD (0.90^*^)	Ensemble using MO and GT features
Karim et al. (2024)	OASIS + SALD (112)	rs-fMRI	CN–AD (0.92 ± 0.02)	SVM using functional connectivity GT metrics
**Our study**	ADNI (394)	sMRI, dMRI	CN–AD (0.94 ± 0.02/0.90 ± 0.05^*^); MCI–AD (0.80 ± 0.06/0.77 ± 0.12^*^); CN–MCI (0.70 ± 0.02/0.65 ± 0.04^*^); LCDP (MSE: 0.53 ± 0.31)	Systematic evaluation of MO, MS, and GT features using NestedCV, diversity-aware Ensembles, and XAI

^*^Balanced accuracy (BACC) is reported where indicated; otherwise, accuracy (ACC) is shown.

EDSD, European DTI Study on Dementia; ADNI, Alzheimer's Disease Neuroimaging Initiative; OASIS, Open Access Series of Imaging Studies; SALD, Southwest University Adult Lifespan Dataset; dMRI, diffusion MRI; sMRI, structural MRI; rs-fMRI, resting-state functional MRI; MO, Morphometric features; MS, Microstructural features; GT, Graph-theoretical features; FA, Fractional Anisotropy; MD, Mean Diffusivity; ACC, Accuracy; BACC, Balanced accuracy; LCDP, Longitudinal cognitive decline prediction.

This systematic analysis highlighted the critical role of multimodal MRI-derived connectivity feature integration. The combination of MO and MS feature sets achieved the best results in CN vs. ADD classification (BACC: 0.898 ± 0.051), while unimodal MS and GT feature sets were weak predictors. Interestingly, for MCI vs. ADD and CN vs. MCI classification tasks, MO features alone provided the highest accuracy (BACC: 0.769 ± 0.116 and 0.652 ± 0.044, respectively), with the addition of MS and GT introducing redundancy. CN vs. MCI remained the most challenging task, underscoring the inherent complexity of detecting early disease stages. Likewise, the ensemble learning provided more robust and stable predictions for the LCDP task, achieving the best overall predictive performance with the MO feature set (RMSE: 0.701 ± 0.221). These findings suggest that integrating multiple learners within an ensemble learning framework helps reduce model-specific errors and improves generalization performance. Also, increasing feature complexity by adding MS and GT features did not lead to further performance improvements. For example, the MO+MS feature set achieved competitive results (RMSE: 0.704 ± 0.230), but it did not outperform the MO feature set alone. Together, these results highlight the dominant contribution of structural brain changes to the prediction of cognitive decline, while suggesting that additional feature modalities may introduce redundancy rather than complementary information.

The XAI analyses (SHAP and LIME) confirmed the biological plausibility of these results. Hippocampal, putamen, thalamus, BPV, and amygdala volumes and amyloid status emerged as consistent predictors across all tasks, with smaller volumes and *Aβ*+ status indicating poorer outcomes. Additional task-specific features included thickness in the temporal pole, parahippocampal regions, and mean diffusivity (MD). These interpretable findings provide insights into the neurobiological underpinnings of disease staging and progression. Together, these results suggest that MO features derived from widely available sMRI are sufficient for robust and clinically meaningful prediction of Alzheimer's disease progression, while multimodal integration adds value in specific contexts. Importantly, the use of XAI methods ensures transparency, facilitating translation into clinical workflows and supporting more accurate and interpretable diagnostic decision-making.

Furthermore, we presented the comparative analysis of the proposed approach with existing related MRI-based ML studies in Alzheimer's disease (Table 4). Most prior studies have focused primarily on individual MRI-derived feature representations (Dyrba et al., 2013; Ebadi et al., 2017; Karim et al., 2024). Even in studies that considered multimodal feature representations, the relative and incremental predictive utility of different feature sets was not systematically evaluated (Diogo et al., 2022). Although these studies demonstrated that individual feature sets (i.e., MO, MS, GT) can represent disease-relevant insights, they were often restricted to cross-sectional diagnostic classification and did not systematically examine the added value of integrating multiple feature representations.

In contrast, the present study provides a systematic evaluation of MO, MS, and GT feature sets, assessed both individually and in combination across multiple clinical tasks. The findings of our study indicated that MO features frequently yield the strongest classification performance, underscoring the continued relevance of regional atrophy as a marker of disease-related cognitive impairment. In contrast, GT features captured meaningful network-level alterations; however, their addition to the MO feature set did not consistently improve predictive performance. These findings are broadly consistent with the observations reported by Diogo et al. (2022). Likewise, the MS feature set derived from dMRI provided complementary information when combined with MO features, but they did not outperform MO feature representation when evaluated independently. Furthermore, by extending the analysis to longitudinal cognitive decline prediction in terms of MMSE score and incorporating XAI methods, our proposed framework advances prior work by addressing both temporal aspects of disease progression and model interpretability. Together, the findings of this study reinforce emerging evidence that structural MO markers retain strong explanatory value, while highlighting the importance of systematically evaluating the trade-off between feature complexity, predictive utility, and interpretability in neuroimaging-based ML models.

### 4.2 Clinical implications

The findings of this study carry significant implications for clinical practice. First, the consistent predictive power of the MO feature set highlights the utility of standard T1w-sMRI, already widely available in routine clinical protocols, as a cost-effective and accessible biomarker source for both staging and prognosis in Alzheimer's disease. Second, the interpretability of the results reinforces their clinical value. On a more general theme, this approach is relevant regarding the identification of the minimum dataset needed to reach meaningful predictions of cognitive outcomes in ADD. This is of relevance regarding the potential translation of the proposed computational approaches to the clinical setting. The integration of XAI ensures that predictive models are transparent and clinically interpretable, allowing AI predictions to complement clinical expertise. Such transparency is essential for the adoption of ML frameworks into clinical workflows, ultimately supporting personalized care and patient stratification.

### 4.3 Limitations and future directions

Building on these findings, we acknowledge several limitations of the present study, which also highlight important future research directions. First, the systematic analysis was conducted using data from the ADNI cohort, although model performance was evaluated using NestedCV, which provides a rigorous internal assessment of stability. Therefore, future research should focus on validating the proposed framework in external independent cohorts, which would further strengthen confidence in the robustness and generalizability of the proposed framework.

Second, this study focused on MO, MS, and GT feature sets that are well established in the neurodegeneration literature, while radiomic features were not included. The radiomic approaches may provide complementary information; therefore, the integration of radiomic features into a multimodal framework represents a promising future research direction. Furthermore, expanding the proposed approach to include complementary biomarkers, such as amyloid- and tau-PET imaging, cerebrospinal fluid and blood biomarkers, could further enhance predictive performance and provide a more comprehensive integration of key metrics able to better capture the complexity of Alzheimer's disease pathology. Particular attention should be directed toward improving early-stage classification, especially the challenging distinction between cognitively normal individuals and those with mild cognitive impairment, by refining feature selection strategies. In the future, this approach could also contribute to predicting treatment response, thereby contributing to the advancement of precision medicine in Alzheimer's disease.

## 5 Conclusion

In conclusion, this ablation experimental analysis demonstrated the effectiveness of MRI-derived connectivity metrics for Alzheimer's disease classification and cognitive decline prediction. The highest performance among all the DSC tasks was reported for the CN vs. the ADD task (BACC: 0.898 ± 0.051) using a combination of MO and MS feature sets with an ensemble learner. However, the MO features alone demonstrated the best performance for MCI vs. ADD (BACC: 0.769 ± 0.116) and CN vs. MCI (BACC: 0.652 ± 0.044), suggesting that the addition of MS and GT features may not enhance classification performance in early disease stages. In the longitudinal cognitive decline prediction task, MO features also yielded the best predictive performance, achieving a lowest MSE of 0.531 ± 0.307 and an R^2^ of 0.212 ± 0.177. These findings highlighted that the integration of additional features provided only marginal gains, which highlights the robustness of sMRI-derived features in modeling disease progression. Furthermore, the findings reported the identification of metric-region significance across various Alzheimer's DSC and LCDP tasks using XAI-based approaches. These interpretable findings of the study provide valuable insights into the association of brain regions with different cognitively impaired stages of AD, supporting clinicians in timely decision-making.

## Data Availability

Publicly available datasets were analyzed in this study. This data can be found here: Data: https://adni.loni.usc.edu/; Code: https://github.com/shahzadali21/MRIConnectADD.

## Footnotes

1
https://adni.loni.usc.edu/

2
https://surfer.nmr.mgh.harvard.edu/

3
https://www.mrtrix.org/

## References

[B1] Aisen P. S. Cummings J. Jack Jr C. R. Morris J. C. Sperling R. Frölich L. . (2017). On the path to 2025: understanding the Alzheimer's disease continuum. Alzheimer's Res. Ther. 9:60. doi: 10.1186/s13195-017-0283-5 28793924 PMC5549378

[B2] Avants B. B. Tustison N. J. Song G. Cook P. A. Klein A. Gee J. C. (2011). A reproducible evaluation of ants similarity metric performance in brain image registration. Neuroimage 54, 2033–2044. doi: 10.1016/j.neuroimage.2010.09.025 20851191 PMC3065962

[B3] Bates S. Hastie T. Tibshirani R. (2024). Cross-validation: what does it estimate and how well does it do it? J. Am. Stat. Assoc. 119, 1434–1445. doi: 10.1080/01621459.2023.2197686 39308484 PMC11412612

[B4] Buckner R. L. Head D. Parker J. Fotenos A. F. Marcus D. Morris J. C. . (2004). A unified approach for morphometric and functional data analysis in young, old, and demented adults using automated atlas-based head size normalization: reliability and validation against manual measurement of total intracranial volume. Neuroimage 23, 724–738. doi: 10.1016/j.neuroimage.2004.06.018 15488422

[B5] Busovaca E. Zimmerman M. E. Meier I. B. Griffith E. Y. Grieve S. M. Korgaonkar M. S. . (2016). Is the Alzheimer's disease cortical thickness signature a biological marker for memory? Brain Imaging Behav. 10, 517–523. doi: 10.1007/s11682-015-9413-5 26040979 PMC4670278

[B6] Dickerson B. C. Bakkour A. Salat D. H. Feczko E. Pacheco J. Greve D. N. . (2009). The cortical signature of Alzheimer's disease: regionally specific cortical thinning relates to symptom severity in very mild to mild ad dementia and is detectable in asymptomatic amyloid-positive individuals. Cerebral Cortex 19, 497–510. doi: 10.1093/cercor/bhn113 18632739 PMC2638813

[B7] Dietterich T. G. (1998). Approximate statistical tests for comparing supervised classification learning algorithms. Neural Comput. 10, 1895–1923. doi: 10.1162/089976698300017197 9744903

[B8] Diogo V. S. Ferreira H. A. Prata D. Initiative A. D. N. (2022). Early diagnosis of Alzheimer's disease using machine learning: a multi-diagnostic, generalizable approach. Alzheimer's Res. Ther. 14:107. doi: 10.1186/s13195-022-01047-y 35922851 PMC9347083

[B9] Dyrba M. Ewers M. Wegrzyn M. Kilimann I. Plant C. Oswald A. . (2013). Robust automated detection of microstructural white matter degeneration in Alzheimer's disease using machine learning classification of multicenter DTI data. PLoS ONE 8:e64925. doi: 10.1371/journal.pone.0064925 23741425 PMC3669206

[B10] Ebadi A. Dalboni da Rocha J. L. Nagaraju D. B. Tovar-Moll F. Bramati I. Coutinho G. . (2017). Ensemble classification of Alzheimer's disease and mild cognitive impairment based on complex graph measures from diffusion tensor images. Front. Neurosci. 11:56. doi: 10.3389/fnins.2017.00056 28293162 PMC5329061

[B11] Elgeldawi E. Sayed A. Galal A. R. Zaki A. M. (2021). “Hyperparameter tuning for machine learning algorithms used for Arabic sentiment analysis,” in Informatics (MDPI), 79. doi: 10.3390/informatics8040079

[B12] Fan C.-C. Yang H. Peng L. Zhou X.-H. Ni Z.-L. Zhou Y.-J. . (2022). Bgl-net: A brain-inspired global-local information fusion network for Alzheimer's disease based on sMRI. IEEE Trans. Cogn. Dev. Syst. 15, 1161–1169. doi: 10.1109/TCDS.2022.3204782

[B13] Feinberg A. (2024). Mrtrix streamlines and tractography. Available online at: https://andysbrainbook.readthedocs.io/en/stable/MRtrix/MRtrix_Course/MRtrix_07_Streamlines.html (Accessed December 29, 2025).

[B14] Fischl B. (2012). Freesurfer. Neuroimage 62, 774–781. doi: 10.1016/j.neuroimage.2012.01.021 22248573 PMC3685476

[B15] Fischl B. Salat D. H. Busa E. Albert M. Dieterich M. Haselgrove C. . (2002). Whole brain segmentation: automated labeling of neuroanatomical structures in the human brain. Neuron 33, 341–355. doi: 10.1016/S0896-6273(02)00569-X 11832223

[B16] Hagberg A. Swart P. J. Schult D. A. (2008). Exploring network structure, dynamics, and function using networkx. Technical report, Los Alamos National Laboratory (LANL), Los Alamos, NM (United States). doi: 10.25080/TCWV9851

[B17] Hassija V. Chamola V. Mahapatra A. Singal A. Goel D. Huang K. . (2024). Interpreting black-box models: a review on explainable artificial intelligence. Cognit. Comput. 16, 45–74. doi: 10.1007/s12559-023-10179-8

[B18] Henriques R. N. Henson R. Cam-CA N. Correia M. M. (2023). Unique information from common diffusion MRI models about white-matter differences across the human adult lifespan. Imaging Neurosci. 1, 1–25. doi: 10.1162/imag_a_00051 40799710 PMC12327084

[B19] Jack Jr C. R. Bernstein M. A. Fox N. C. Thompson P. Alexander G. Harvey D. . (2008). The Alzheimer's disease neuroimaging initiative (Adni): MRI methods. J. Magn. Reson. Imag. 27, 685–691. doi: 10.1002/jmri.21049 18302232 PMC2544629

[B20] Karim S. S. Fahad M. S. Rathore R. (2024). Identifying discriminative features of brain network for prediction of Alzheimer's disease using graph theory and machine learning. Front. Neuroinform. 18:1384720. doi: 10.3389/fninf.2024.1384720 38957548 PMC11217540

[B21] Lane C. A. Hardy J. Schott J. M. (2018). Alzheimer's disease. Eur. J. Neurol. 25, 59–70. doi: 10.1111/ene.13439 28872215

[B22] Liu M. Zhang H. Shi F. Shen D. (2023). Hierarchical graph convolutional network built by multiscale atlases for brain disorder diagnosis using functional connectivity. IEEE Trans. Neural Netw. Learn. Syst. 35, 15182–15194. doi: 10.1109/TNNLS.2023.3282961 37339027

[B23] Madole J. W. Buchanan C. R. Rhemtulla M. Ritchie S. J. Bastin M. E. Deary I. J. . (2023). Strong intercorrelations among global graph-theoretic indices of structural connectivity in the human brain. Neuroimage 275:120160. doi: 10.1016/j.neuroimage.2023.120160 37169117

[B24] Oxtoby N. P. Garbarino S. Firth N. C. Warren J. D. Schott J. M. Alexander D. C. . (2017). Data-driven sequence of changes to anatomical brain connectivity in sporadic Alzheimer's disease. Front. Neurol. 8:580. doi: 10.3389/fneur.2017.00580 29163343 PMC5681907

[B25] Patenaude B. Smith S. M. Kennedy D. N. Jenkinson M. (2011). A Bayesian model of shape and appearance for subcortical brain segmentation. Neuroimage 56, 907–922. doi: 10.1016/j.neuroimage.2011.02.046 21352927 PMC3417233

[B26] Phillips D. J. McGlaughlin A. Ruth D. Jager L. R. Soldan A. Initiative A. D. N. . (2015). Graph theoretic analysis of structural connectivity across the spectrum of Alzheimer's disease: the importance of graph creation methods. NeuroImage 7, 377–390. doi: 10.1016/j.nicl.2015.01.007 25984446 PMC4429220

[B27] Probst P. Boulesteix A.-L. Bischl B. (2019). Tunability: Importance of hyperparameters of machine learning algorithms. J. Mach. Learn. Res. 20, 1–32. doi: 10.48550/arXiv.1802.09596

[B28] Ribeiro M. T. Singh S. Guestrin C. (2016). “‘Why should i trust you?' explaining the predictions of any classifier,” in Proceedings of the 22nd ACM SIGKDD International Conference on Knowledge Discovery and Data Mining, 1135–1144. doi: 10.1145/2939672.2939778

[B29] Robinson L. Tang E. Taylor J.-P. (2015). Dementia: timely diagnosis and early intervention. BMJ 350:h3029. doi: 10.1136/bmj.h3029 26079686 PMC4468575

[B30] Rubinov M. Sporns O. (2010). Complex network measures of brain connectivity: uses and interpretations. Neuroimage 52, 1059–1069. doi: 10.1016/j.neuroimage.2009.10.003 19819337

[B31] Salih A. M. Raisi-Estabragh Z. Galazzo I. B. Radeva P. Petersen S. E. Lekadir K. . (2025). A perspective on explainable artificial intelligence methods: SHAP and lime. Adv. Intell. Syst. 7:2400304. doi: 10.1002/aisy.202400304

[B32] Smith R. E. Tournier J.-D. Calamante F. Connelly A. (2015). Sift2: Enabling dense quantitative assessment of brain white matter connectivity using streamlines tractography. Neuroimage 119, 338–351. doi: 10.1016/j.neuroimage.2015.06.092 26163802

[B33] Smith S. M. (2002). Fast robust automated brain extraction. Hum. Brain Mapp. 17, 143–155. doi: 10.1002/hbm.10062 12391568 PMC6871816

[B34] Smith S. M. Jenkinson M. Woolrich M. W. Beckmann C. F. Behrens T. E. Johansen-Berg H. . (2004). Advances in functional and structural MR image analysis and implementation as FSL. Neuroimage 23, S208–S219. doi: 10.1016/j.neuroimage.2004.07.051 15501092

[B35] Snoek L. Miletić S. Scholte H. S. (2019). How to control for confounds in decoding analyses of neuroimaging data. Neuroimage 184, 741–760. doi: 10.1016/j.neuroimage.2018.09.074 30268846

[B36] Tae W.-S. Ham B.-J. Pyun S.-B. Kim B.-J. (2025). Current clinical applications of structural mri in neurological disorders. J. Clin. Neurol. 21:277. doi: 10.3988/jcn.2025.0185 40635533 PMC12303675

[B37] Tahedl M. (2018). BATMAN: basic and advanced tractography with MRtrix for all neurophiles. OSF. doi: 10.17605/OSF.IO/10.17605

[B38] Tournier J.-D. Smith R. Raffelt D. Tabbara R. Dhollander T. Pietsch M. . (2019). Mrtrix3: a fast, flexible and open software framework for medical image processing and visualisation. Neuroimage 202:116137. doi: 10.1016/j.neuroimage.2019.116137 31473352

[B39] Varma S. Simon R. (2006). Bias in error estimation when using cross-validation for model selection. BMC Bioinform. 7:91. doi: 10.1186/1471-2105-7-91 16504092 PMC1397873

[B40] Whitwell J. L. Crum W. R. Watt H. C. Fox N. C. (2001). Normalization of cerebral volumes by use of intracranial volume: implications for longitudinal quantitative MR imaging. Am. J. Neuroradiol. 22, 1483–1489. 11559495 PMC7974589

[B41] Wilcoxon F. (1945). Individual comparisons by ranking methods. Biometr. Bull. 1, 80–83. doi: 10.2307/3001968

[B42] Zhang Y. Brady M. Smith S. (2001). Segmentation of brain MR images through a hidden markov random field model and the expectation-maximization algorithm. IEEE Trans. Med. Imaging 20, 45–57. doi: 10.1109/42.906424 11293691

[B43] Zhang Y. He X. Chan Y. H. Teng Q. Rajapakse J. C. (2023). Multi-modal graph neural network for early diagnosis of Alzheimer's disease from sMRI and pet scans. Comput. Biol. Med. 164:107328. doi: 10.1016/j.compbiomed.2023.107328 37573721

[B44] Zhang Y. Xue L. Zhang S. Yang J. Zhang Q. Wang M. . (2024). A novel spatiotemporal graph convolutional network framework for functional connectivity biomarkers identification of Alzheimer's disease. Alzheimer's Res. Ther. 16:60. doi: 10.2139/ssrn.4469475 38481280 PMC10938710

