# Supplementary material for: A systematic study on the integration of MRI connectivity metrics for Alzheimer's diagnosis, staging, and cognitive decline prediction
Source: Front Neuroimaging. 2026 Feb 17;5:1746464. doi: 10.3389/fnimg.2026.1746464 (PMC12953077; doi:10.3389/fnimg.2026.1746464)
Supplement: Supplementary file 1 [file Data_Sheet_1.pdf]

Supplementary Material

A systematic study on the integration of MRI connectivity metrics for Alzheimer's diagnosis, staging, and cognitive decline prediction

Supplementary Table S1 || The list of 84 anatomical brain regions of interest (ROIs) in Desikan-Killiany atlas with the short labels of brain regions (nodes).

Node	Short	DK atlas brain regions	Node	Short	DK atlas brain regions
**1**	L.BSTS	ctx-lh-bankssts	**43**	R.TH	Right-Thalamus
**2**	L.CACG	ctx-lh-caudalanteriorcingulate	**44**	R.CA	Right-Caudate
**3**	L.CMFG	ctx-lh-caudalmiddlefrontal	**45**	R.PU	Right-Putamen
**4**	L.CU	ctx-lh-cuneus	**46**	R.PA	Right-Pallidum
**5**	L.EC	ctx-lh-entorhinal	**47**	R.HI	Right-Hippocampus
**6**	L.FG	ctx-lh-fusiform	**48**	R.AM	Right-Amygdala
**7**	L.IPG	ctx-lh-inferiorparietal	**49**	R.AC	Right-Accumbens-area
**8**	L.ITG	ctx-lh-inferiortemporal	**50**	R.BSTS	ctx-rh-bankssts
**9**	L.ICG	ctx-lh-isthmuscingulate	**51**	R.CACG	ctx-rh-caudalanteriorcingulate
**10**	L.LOG	ctx-lh-lateraloccipital	**52**	R.CMFG	ctx-rh-caudalmiddlefrontal
**11**	L.LOFG	ctx-lh-lateralorbitofrontal	**53**	R.CU	ctx-rh-cuneus
**12**	L.LG	ctx-lh-lingual	**54**	R.EC	ctx-rh-entorhinal
**13**	L.MOFG	ctx-lh-medialorbitofrontal	**55**	R.FG	ctx-rh-fusiform
**14**	L.MTG	ctx-lh-middletemporal	**56**	R.IPG	ctx-rh-inferiorparietal
**15**	L.PHIG	ctx-lh-parahippocampal	**57**	R.ITG	ctx-rh-inferiortemporal
**16**	L.PaCG	ctx-lh-paracentral	**58**	R.ICG	ctx-rh-isthmuscingulate
**17**	L.POP	ctx-lh-parsopercularis	**59**	R.LOG	ctx-rh-lateraloccipital
**18**	L.POR	ctx-lh-parsorbitalis	**60**	R.LOFG	ctx-rh-lateralorbitofrontal
**19**	L.PTR	ctx-lh-parstriangularis	**61**	R.LG	ctx-rh-lingual
**20**	L.PCAL	ctx-lh-pericalcarine	**62**	R.MOFG	ctx-rh-medialorbitofrontal
**21**	L.PoCG	ctx-lh-postcentral	**63**	R.MTG	ctx-rh-middletemporal
**22**	L.PCG	ctx-lh-posteriorcingulate	**64**	R.PHIG	ctx-rh-parahippocampal
**23**	L.PrCG	ctx-lh-precentral	**65**	R.PaCG	ctx-rh-paracentral
**24**	L.PCU	ctx-lh-precuneus	**66**	R.POP	ctx-rh-parsopercularis
**25**	L.RACG	ctx-lh-rostralanteriorcingulate	**67**	R.POR	ctx-rh-parsorbitalis
**26**	L.RMFG	ctx-lh-rostralmiddlefrontal	**68**	R.PTR	ctx-rh-parstriangularis
**27**	L.SFG	ctx-lh-superiorfrontal	**69**	R.PCAL	ctx-rh-pericalcarine
**28**	L.SPG	ctx-lh-superiorparietal	**70**	R.PoCG	ctx-rh-postcentral
**29**	L.STG	ctx-lh-superiortemporal	**71**	R.PCG	ctx-rh-posteriorcingulate
**30**	L.SMG	ctx-lh-supramarginal	**72**	R.PrCG	ctx-rh-precentral

**31**	L.FP	ctx-lh-frontalpole	**73**	R.PCU	ctx-rh-precuneus
**32**	L.TP	ctx-lh-temporalpole	**74**	R.RACG	ctx-rh-rostralanteriorcingulate
**33**	L.TTG	ctx-lh-transversetemporal	**75**	R.RMFG	ctx-rh-rostralmiddlefrontal
**34**	L.IN	ctx-lh-insula	**76**	R.SFG	ctx-rh-superiorfrontal
**35**	L.CER	Left-Cerebellum-Cortex	**77**	R.SPG	ctx-rh-superiorparietal
**36**	L.TH	Left-Thalamus	**78**	R.STG	ctx-rh-superiortemporal
**37**	L.CA	Left-Caudate	**79**	R.SMG	ctx-rh-supramarginal
**38**	L.PU	Left-Putamen	**80**	R.FP	ctx-rh-frontalpole
**39**	L.PA	Left-Pallidum	**81**	R.TP	ctx-rh-temporalpole
**40**	L.HI	Left-Hippocampus	**82**	R.TTG	ctx-rh-transversetemporal
**41**	L.AM	Left-Amygdala	**83**	R.IN	ctx-rh-insula
**42**	L.AC	Left-Accumbens-area	**84**	R.CER	Right-Cerebellum-Cortex

Supplementary Table S2 || a complete list of the global and nodal graph-theoretic (GT) features extracted from subject-specific structural brain networks, together with their abbreviations, definitions, and references. Brain networks were constructed using 82 cortical and subcortical regions defined by the Desikan–Killiany atlas, where nodes correspond to brain regions and edges represent tractography-derived white matter connections.

**Sr.**	**Global graph-theoretic (GT) metrics**		**Sr.**	**Nodal graph-theoretic (GT) metrics**	
	**Feature Name**	**Abbreviation Used**		**Feature Name**	**Abbreviation Used**
**1**	Network density	GT_G_Density	**1**	Degree centrality (node i)	GT_N_DC_i
**2**	Modularity	GT_G_Modularity	**2**	Clustering coefficient (node i)	GT_N_CC_i
**3**	Assortativity	GT_G_Assortativity	**3**	Betweenness centrality (node i)	GT_N_BC_i
**4**	Transitivity	GT_G_Transitivity	**4**	Eigenvector centrality (node i)	GT_N_EC_i
**5**	Global efficiency	GT_G_Efficiency	**5**	Closeness centrality (node i)	GT_N_CClo_i
**6**	Characteristic path length	GT_G_CPL	**6**	Node strength (node i)	GT_N_NS_i
**7**	Network diameter	GT_G_Diameter	**7**	PageRank (node i)	GT_N_PR_i
**8**	Degree distribution entropy	GT_G_DegEnt			
**9**	Spectral radius	GT_G_SpecRad			
**10**	Average clustering coefficient	GT_G_AvgCC			
**11**	Small-worldness	GT_G_SW			
**12**	Average degree	GT_G_avgDeg			
**13**	Average betweenness centrality	GT_G_avgBC			
**14**	Average eigenvector centrality	GT_G_avgEC			
**15**	Average closeness centrality	GT_G_avgCClo			
**16**	Average node strength	GT_G_avgNS			
**17**	Average PageRank	GT_G_avgPR			

GT: graph-theoretic measures; **G**: global (whole-network) metrics; **N**: nodal (local) metrics. For nodal measures, i refers to the node index (i = 0–81), corresponding to brain regions defined by the DK atlas (excluding (Left/Right)-Cerebellum-Cortex). Each nodal metric was computed independently for all nodes using the same definition. Abbreviations are used consistently across figures and tables to improve readability.

Supplementary Table S3 || Hyperparameter grids used for model optimization.

A) Hyperparameter grids explored for machine learning models used in the Alzheimer's disease cognitive stage classification (DSC) tasks.

Model	Hyperparameter grid explored
**Logistic Regression (LogR)**	***Penalty***: L1, L2, ElasticNet; C: 0.001, 0.01, 0.1, 1, 10, 100; ***L1 ratio***: 0.25, 0.5, 0.75; ***Class weight***: None, Balanced; ***Max iterations***: 2000–3000
**SGD Classifier (LR-SGD)**	***Loss***: log-loss; ***Penalty***: L1, L2, ElasticNet; ***Alpha***: 1e-5–1e-2; ***Learning rate***: optimal, adaptive; ***Initial learning rate*** (η_0): 0.001, 0.01; ***Max iterations***: 2000
**Linear Discriminant Analysis (LDA)**	***Solver***: lsqr; ***Shrinkage***: auto, 0–1; ***Tolerance***: 1e-4, 1e-3, 1e-2
**Decision Tree (DT)**	***Criterion***: gini, entropy; ***Max depth***: 3, 5, 10, 15, 20, None; ***Min samples split***: 2, 5, 10; ***Min samples leaf***: 1, 2, 4; ***Max features***: 0.3, 0.5, 0.7, 1.0
**Random Forest (RF)**	***Number of trees***: 50, 100, 200; ***Max depth***: 5, 10, 20, None; ***Min samples split***: 2, 5; ***Min samples leaf***: 1, 2; ***Bootstrap***: True, False; ***Max features***: sqrt, log2
**AdaBoost (AdaBoost)**	***Number of estimators***: 50, 100, 200; ***Learning rate***: 0.01, 0.1, 0.5, 1.0; ***Base estimator depth***: 1, 2
**Support Vector Machine (SVM)**	***Kernel***: linear, polynomial, RBF; ***C***: 0.1, 1, 10, 100; ***Degree***: 2, 3; ***Gamma***: scale, auto
**Extreme Gradient Boosting (XGB)**	***Number of estimators***: 50, 100, 200; ***Learning rate***: 0.01, 0.1, 0.3; ***Max depth***: 3, 5, 7; ***Subsample***: 0.8, 1.0; ***Column sample by tree***: 0.8, 1.0; ***Gamma***: 0, 0.1; ***L1 regularization***: 0, 0.1; ***L2 regularization***: 1, 1.5; ***Class weight scaling***: 1, 2, 5

B) Hyperparameter grids explored for machine learning models used in the longitudinal cognitive decline prediction (LCDP) task.

Model	Hyperparameter grid explored
**Linear Regression (LR)**	***Fit intercept***: True, False
**Ridge Regression**	***Alpha***: 0.001, 0.01, 0.1, 1, 10, 100
**Lasso Regression**	***Alpha***: 0.001, 0.01, 0.1, 1, 10, 100
**ElasticNet Regression**	***Alpha***: 0.001, 0.01, 0.1, 1; L1 ratio: 0.1, 0.5, 0.7, 1.0
**Decision Tree Regressor (DT)**	***Max depth***: 3, 5, 10, 15, 20, None; ***Min samples split***: 2, 5, 10; ***Max features***: 0.3, 0.5, 0.7, 1.0
**Random Forest Regressor (RF)**	***Number of trees***: 10, 50, 100; ***Max depth:*** 3, 5, 10, 15, 20, None; ***Min samples split***: 2, 5, 10; ***Min samples leaf***: 1, 2, 4; ***Bootstrap***: True, False
**AdaBoost Regressor**	***Number of estimators***: 10, 50, 100; ***Learning rate***: 0.01, 0.1, 0.5, 1.0
**Support Vector Regressor (SVR)**	***Kernel***: linear, polynomial, RBF, sigmoid; ***C***: 0.01, 0.1, 1, 10, 100; ***Epsilon***: 0.01, 0.1, 1
**Gaussian Process Regressor (GPR)**	***Kernel***: RBF, Matérn (ν=1.5), Rational Quadratic, Dot Product; ***Alpha***: 1e-10, 1e-5, 1e-2; ***Optimizer restarts***: 0, 5, 10
**Extreme Gradient Boosting Regressor (XGB)**	***Number of estimators***: 10, 50, 100; ***Learning rate***: 0.01, 0.1, 0.3; ***Max depth***: 3, 5, 7; ***Min child weight***: 1, 3, 5; ***Subsample***: 0.8, 1.0; ***Column sample by tree***: 0.8, 1.0; ***Gamma***: 0, 0.1, 0.2; ***L1 regularization***: 0, 0.1, 0.5; ***L2 regularization***: 1, 1.5, 2

Note: Hyperparameter optimization was performed independently for each model using grid search within a nested cross-validation framework.

Supplementary Table S4_A || Performance comparison of base classifiers and ensemble using individual and combination of feature sets for the CN—ADD task.

FeatureSet	Model	ACC	BACC	SEN	SPE	rocAUC
MO	**AdaBoost**	0.901±0.016	0.751±0.030	0.524±0.070	0.978±0.022	0.917±0.067
	**DT**	0.879±0.041	0.778±0.080	0.627±0.155	0.930±0.039	0.783±0.079
	**LDA**	0.912±0.024	0.859±0.056	0.780±0.114	0.939±0.024	0.949±0.039
	**LogR**	0.905±0.024	**0.872±0.056**	**0.822±0.127**	0.921±0.033	0.947±0.042
	**RF**	***0.931±0.027***	0.836±0.054	0.693±0.099	0.978±0.022	0.937±0.058
	**SVM**	0.887±0.052	0.844±0.045	0.780±0.083	0.907±0.064	0.936±0.039
	**XGB**	0.905±0.027	0.777±0.052	0.584±0.102	0.969±0.029	0.930±0.042
	**Ensemble**	**0.942±0.024***	0.868±0.052	0.758±0.097	**0.978±0.015**	**0.951±0.034**
**FeatureSet**	**Model**	**ACC**	**BACC**	**SEN**	**SPE**	**rocAUC**
MS	**AdaBoost**	0.854±0.029	0.650±0.065	0.344±0.127	0.956±0.022	0.821±0.052
	**DT**	0.781±0.033	0.719±0.083	0.627±0.234	0.812±0.078	0.748±0.060
	**LDA**	**0.861±0.028**	0.636±0.087	0.298±0.186	**0.974±0.029**	0.846±0.052
	**LogR**	0.756±0.057	0.757±0.046	0.760±0.053	0.754±0.066	0.807±0.067
	**RF**	0.825±0.059	0.710±0.122	0.538±0.259	0.882±0.069	0.838±0.079
	**SVM**	0.723±0.109	**0.773±0.097**	**0.849±0.096**	0.697±0.117	0.839±0.072
	**XGB**	0.781±0.070	0.693±0.085	0.562±0.164	0.824±0.083	0.804±0.059
	**Ensemble**	0.814±0.029	**0.774±0.025**	0.716±0.067	0.833±0.042	**0.860±0.042**
**FeatureSet**	**Model**	**ACC**	**BACC**	**SEN**	**SPE**	**rocAUC**
GT	**AdaBoost**	0.821±0.023	0.561±0.041	0.171±0.087	0.952±0.028	0.832±0.059
	**DT**	0.723±0.052	0.627±0.051	0.482±0.158	0.773±0.089	0.665±0.050
	**LDA**	0.825±0.111	0.738±0.097	0.607±0.106	0.869±0.124	0.832±0.126
	**LogR**	**0.872±0.027**	0.811±0.065	0.718±0.125	0.903±0.012	**0.902±0.036**
	**RF**	0.828±0.025	0.567±0.051	0.173±0.097	**0.960±0.019**	0.770±0.105
	**SVM**	0.843±0.113	0.776±0.093	0.673±0.208	0.878±0.152	0.850±0.101
	**XGB**	0.803±0.036	0.596±0.061	0.284±0.171	0.908±0.068	0.781±0.074
	**Ensemble**	**0.872±0.054**	**0.820±0.097**	**0.740±0.166**	0.899±0.036	0.900±0.052
**FeatureSet**	**Model**	**ACC**	**BACC**	**SEN**	**SPE**	**rocAUC**
MO+MS	**AdaBoost**	0.905±0.015	0.762±0.041	0.547±0.095	0.978±0.022	0.934±0.037
	**DT**	0.880±0.058	0.762±0.089	0.584±0.151	0.939±0.057	0.766±0.184
	**LDA**	0.916±0.033	0.835±0.049	0.713±0.131	0.956±0.054	0.946±0.027
	**LogR**	0.909±0.018	0.856±0.074	0.778±0.176	0.934±0.035	0.934±0.068
	**RF**	**0.927±0.026**	0.807±0.057	0.627±0.108	**0.987±0.019**	0.924±0.054
	**SVM**	0.876±0.052	0.818±0.081	0.733±0.186	0.903±0.072	0.919±0.045
	**XGB**	0.905±0.039	0.758±0.093	0.538±0.173	0.978±0.022	0.925±0.052
	**Ensemble**	**0.920±0.030**	**0.898±0.051***	**0.867±0.093***	0.930±0.028	**0.959±0.029***

FeatureSet	Model	ACC	BACC	SEN	SPE	rocAUC
MO+GT	AdaBoost	0.901±0.017	0.767±0.040	0.564±0.081	0.969±0.019	0.891±0.080
	DT	0.883±0.017	0.766±0.051	0.589±0.134	0.943±0.039	0.760±0.060
	LDA	0.916±0.036	0.808±0.102	0.647±0.202	0.969±0.012	0.938±0.038
	LogR	**0.923±0.039**	0.857±0.069	0.758±0.125	0.956±0.034	0.925±0.035
	RF	0.909±0.026	0.734±0.086	0.473±0.175	**0.996±0.010***	0.931±0.045
	SVM	0.901±0.038	0.793±0.073	0.629±0.130	0.956±0.027	0.896±0.025
	XGB	0.905±0.037	0.777±0.063	0.584±0.102	0.970±0.029	0.902±0.093
	Ensemble	0.916±0.033	**0.887±0.060**	**0.844±0.127**	0.930±0.039	**0.946±0.039**
**FeatureSet**	**Model**	**ACC**	**BACC**	**SEN**	**SPE**	**rocAUC**
MS+GT	AdaBoost	0.839±0.033	0.616±0.057	0.280±0.114	0.952±0.036	0.854±0.058
	DT	0.781±0.080	0.694±0.116	0.562±0.198	0.825±0.079	0.691±0.101
	LDA	0.879±0.045	0.778±0.077	0.627±0.134	0.930±0.037	0.883±0.076
	LogR	0.872±0.048	0.802±0.061	0.696±0.092	0.908±0.047	0.908±0.043
	RF	0.847±0.028	0.604±0.072	0.238±0.140	**0.969±0.020**	0.786±0.105
	SVM	0.850±0.115	0.789±0.073	0.696±0.164	0.882±0.154	0.858±0.101
	XGB	0.814±0.038	0.654±0.098	0.413±0.213	0.895±0.052	0.733±0.094
	Ensemble	**0.883±0.028**	**0.844±0.053**	**0.784±0.104**	0.904±0.029	**0.908±0.047**
**FeatureSet**	**Model**	**ACC**	**BACC**	**SEN**	**SPE**	**rocAUC**
MO+MS+GT	AdaBoost	0.909±0.019	0.771±0.040	0.564±0.081	0.978±0.022	0.879±0.083
	DT	0.901±0.040	0.792±0.069	0.627±0.134	0.956±0.043	0.806±0.048
	LDA	0.923±0.040	0.830±0.104	0.691±0.202	0.969±0.019	0.943±0.034
	LogR	**0.931±0.050**	0.853±0.078	0.736±0.130	0.969±0.045	0.920±0.065
	RF	0.912±0.024	0.756±0.065	0.520±0.132	**0.991±0.019**	0.943±0.038
	SVM	0.909±0.037	0.805±0.079	0.649±0.150	0.961±0.028	0.908±0.026
	XGB	0.909±0.036	0.796±0.075	0.627±0.134	0.965±0.025	0.908±0.081
	Ensemble	0.894±0.036	**0.866±0.057**	**0.824±0.101**	0.908±0.033	**0.946±0.039**

Note: All results are reported as mean±standard deviation, computed from NestedCV.
Within each feature set, the best-performing model is **bolded**. The overall best result (across all models and feature sets) is both **bolded** and marked with an asterisk *** (bolded*)**.

Supplementary Table S4_B || Performance comparison of base classifiers and ensemble using individual and combination of feature sets for the MCI—ADD task.

FeatureSet	Model	ACC	BACC	SEN	SPE	rocAUC
MO	**AdaBoost**	0.771±0.076	0.681±0.086	0.478±0.145	0.883±0.090	0.797±0.098
	**DT**	0.735±0.068	0.690±0.073	0.589±0.108	0.792±0.072	0.695±0.076
	**LDA**	0.729±0.101	0.701±0.144	0.636±0.274	0.767±0.096	0.792±0.088
	**LogR**	0.747±0.101	0.726±0.136	0.678±0.224	0.775±0.070	0.822±0.091
	**RF**	0.777±0.063	0.693±0.075	0.502±0.134	**0.883±0.075**	0.823±0.092
	**SVM**	0.735±0.099	0.697±0.121	0.611±0.176	0.783±0.080	0.773±0.113
	**XGB**	**0.783±0.084**	0.743±0.072	0.653±0.084	0.833±0.106	0.785±0.087
	**Ensemble**	0.771±0.097	**0.769±0.116***	**0.762±0.179***	0.775±0.091	**0.832±0.085***
**FeatureSet**	**Model**	**ACC**	**BACC**	**SEN**	**SPE**	**rocAUC**
MS	**AdaBoost**	0.753±0.047	0.582±0.087	0.198±0.184	**0.967±0.019**	0.611±0.086
	**DT**	0.591±0.071	0.524±0.105	0.382±0.287	0.667±0.141	0.527±0.119
	**LDA**	**0.759±0.049**	0.605±0.054	0.260±0.095	0.950±0.068	0.714±0.068
	**LogR**	0.620±0.075	0.631±0.079	0.653±0.139	0.608±0.096	0.686±0.097
	**RF**	0.723±0.026	0.582±0.055	0.264±0.152	0.900±0.063	0.667±0.053
	**SVM**	0.602±0.112	0.644±0.062	**0.738±0.102**	0.550±0.180	0.716±0.073
	**XGB**	0.698±0.040	0.557±0.074	0.240±0.200	0.875±0.093	0.616±0.114
	**Ensemble**	0.674±0.094	**0.661±0.055**	0.631±0.051	0.692±0.146	**0.735±0.056**
**FeatureSet**	**Model**	**ACC**	**BACC**	**SEN**	**SPE**	**rocAUC**
GT	**AdaBoost**	**0.729±0.022**	0.593±0.061	0.287±0.171	0.900±0.063	0.674±0.051
	**DT**	0.675±0.067	0.607±0.060	0.456±0.114	0.758±0.099	0.585±0.051
	**LDA**	0.669±0.064	0.591±0.066	0.416±0.151	0.767±0.105	0.662±0.122
	**LogR**	0.705±0.078	0.651±0.094	0.527±0.175	0.775±0.096	0.714±0.046
	**RF**	0.723±0.023	0.527±0.038	0.087±0.092	**0.967±0.035***	0.642±0.066
	**SVM**	0.717±0.052	0.622±0.089	0.411±0.191	0.833±0.059	0.619±0.117
	**XGB**	0.674±0.067	0.561±0.040	0.304±0.092	0.817±0.120	0.573±0.072
	**Ensemble**	0.698±0.059	**0.699±0.026**	**0.698±0.078**	0.700±0.108	**0.732±0.067**
**FeatureSet**	**Model**	**ACC**	**BACC**	**SEN**	**SPE**	**rocAUC**
MO+MS	**AdaBoost**	0.771±0.070	0.681±0.083	0.478±0.145	0.883±0.080	0.795±0.105
	**DT**	0.777±0.102	0.707±0.104	0.547±0.146	0.867±0.116	0.750±0.083
	**LDA**	0.711±0.096	0.689±0.141	0.636±0.274	0.742±0.090	0.782±0.099
	**LogR**	0.735±0.103	0.690±0.148	0.589±0.259	0.792±0.066	0.809±0.104
	**RF**	0.789±0.057	0.693±0.089	0.478±0.165	**0.908±0.035**	0.799±0.105
	**SVM**	0.735±0.089	0.703±0.113	0.631±0.165	0.775±0.063	0.791±0.109
	**XGB**	**0.795±0.058***	**0.753±0.088**	0.656±0.169	0.850±0.048	0.781±0.078
	**Ensemble**	0.759±0.107	0.741±0.127	**0.698±0.175**	0.783±0.085	**0.817±0.093**

FeatureSet	Model	ACC	BACC	SEN	SPE	rocAUC
MO+GT	AdaBoost	0.747±0.073	0.659±0.101	0.460±0.189	0.858±0.070	0.783±0.041
	DT	0.741±0.067	0.700±0.080	0.609±0.124	0.792±0.066	0.698±0.084
	LDA	0.759±0.036	0.686±0.051	0.522±0.093	0.850±0.023	0.783±0.092
	LogR	0.765±0.091	0.717±0.113	0.609±0.184	0.825±0.085	0.769±0.125
	RF	**0.783±0.050**	0.662±0.064	0.391±0.096	**0.933±0.037**	0.788±0.067
	SVM	0.711±0.029	0.598±0.048	0.347±0.115	0.850±0.048	0.721±0.094
	XGB	0.735±0.065	0.652±0.077	0.462±0.173	0.842±0.095	0.775±0.096
	Ensemble	0.753±0.069	**0.729±0.093**	**0.676±0.170**	0.783±0.068	**0.805±0.064**
**FeatureSet**	**Model**	**ACC**	**BACC**	**SEN**	**SPE**	**rocAUC**
MS+GT	AdaBoost	**0.741±0.072**	0.617±0.099	0.333±0.236	0.900±0.109	0.725±0.073
	DT	0.615±0.064	0.559±0.027	0.436±0.177	0.683±0.149	0.538±0.056
	LDA	0.675±0.062	0.595±0.059	0.416±0.151	0.775±0.109	0.684±0.088
	LogR	0.692±0.080	0.653±0.097	0.564±0.177	0.742±0.095	0.696±0.059
	RF	0.711±0.050	0.512±0.058	0.067±0.099	**0.958±0.059**	0.622±0.047
	SVM	0.602±0.191	0.586±0.086	0.547±0.299	0.625±0.358	0.622±0.142
	XGB	0.687±0.078	0.544±0.100	0.222±0.176	0.867±0.068	0.569±0.039
	Ensemble	0.705±0.069	**0.697±0.069**	**0.678±0.159**	0.717±0.112	**0.758±0.081**
**FeatureSet**	**Model**	**ACC**	**BACC**	**SEN**	**SPE**	**rocAUC**
MO+MS+GT	AdaBoost	0.741±0.050	0.655±0.043	0.460±0.105	0.850±0.086	0.805±0.089
	DT	0.687±0.067	0.646±0.115	0.551±0.244	0.742±0.054	0.641±0.127
	LDA	0.747±0.067	0.684±0.072	0.542±0.098	0.825±0.068	0.742±0.092
	LogR	0.759±0.090	0.707±0.116	0.589±0.191	0.825±0.075	0.771±0.124
	RF	**0.777±0.049**	0.639±0.075	0.329±0.141	**0.950±0.019**	0.807±0.079
	SVM	0.735±0.054	0.643±0.085	0.436±0.177	0.850±0.056	0.726±0.089
	XGB	0.741±0.049	0.663±0.078	0.484±0.177	0.842±0.062	0.768±0.105
	Ensemble	0.759±0.043	**0.754±0.050**	**0.742±0.137**	0.767±0.081	**0.825±0.053**

Note: All results are reported as mean±standard deviation, computed from NestedCV.
Within each feature set, the best-performing model is **bolded**. The overall best result (across all models and feature sets) is both **bolded** and marked with an asterisk *** (bolded*)**.

Supplementary Table S4_C || Performance comparison of base classifiers and ensemble using individual and combination of feature sets for the CN—MCI task.

FeatureSet	Model	ACC	BACC	SEN	SPE	rocAUC
MO	AdaBoost	0.678±0.034	0.579±0.030	0.258±0.062	**0.899±0.060***	0.631±0.076
	DT	0.643±0.061	0.617±0.073	0.533±0.249	0.700±0.166	0.621±0.106
	LDA	0.687±0.068	0.645±0.076	0.508±0.119	0.781±0.069	**0.687±0.102**
	LogR	0.647±0.072	0.631±0.082	0.583±0.132	0.680±0.066	0.674±0.102
	RF	0.701±0.067	0.626±0.080	0.383±0.173	0.869±0.099	0.672±0.077
	SVM	**0.704±0.029***	**0.652±0.044***	0.483±0.096	0.820±0.019	0.656±0.093
	XGB	0.681±0.045	0.610±0.064	0.383±0.170	0.837±0.082	0.635±0.111
	Ensemble	0.647±0.054	0.628±0.072	**0.567±0.155**	0.689±0.064	**0.687±0.104**
**FeatureSet**	**Model**	**ACC**	**BACC**	**SEN**	**SPE**	**rocAUC**
MS	AdaBoost	0.672±0.024	0.631±0.037	0.500±0.128	0.763±0.073	0.660±0.030
	DT	0.635±0.047	0.610±0.070	0.533±0.209	0.688±0.110	0.645±0.057
	LDA	**0.690±0.063**	0.599±0.062	0.308±0.096	**0.890±0.083**	0.683±0.093
	LogR	0.632±0.082	0.636±0.088	**0.650±0.166**	0.622±0.117	0.685±0.095
	RF	0.658±0.032	0.624±0.046	0.517±0.109	0.732±0.045	0.666±0.034
	SVM	0.638±0.082	**0.639±0.085**	0.642±0.146	0.636±0.112	0.683±0.089
	XGB	0.647±0.025	0.624±0.018	0.550±0.099	0.697±0.082	0.677±0.025
	Ensemble	0.618±0.076	0.623±0.082	0.642±0.181	0.605±0.126	**0.691±0.085**
**FeatureSet**	**Model**	**ACC**	**BACC**	**SEN**	**SPE**	**rocAUC**
GT	AdaBoost	0.658±0.042	0.573±0.046	0.300±0.123	**0.846±0.083**	0.605±0.024
	DT	0.635±0.069	0.569±0.065	0.358±0.109	0.780±0.101	0.561±0.047
	LDA	0.612±0.062	0.575±0.068	0.458±0.125	0.693±0.082	0.586±0.094
	LogR	0.627±0.046	0.585±0.074	0.450±0.168	0.719±0.032	0.596±0.108
	RF	**0.661±0.035**	0.585±0.050	0.342±0.119	0.829±0.047	0.634±0.058
	SVM	0.629±0.041	**0.599±0.045**	0.500±0.083	0.697±0.055	0.613±0.062
	XGB	0.589±0.031	0.562±0.023	0.475±0.120	0.650±0.099	0.608±0.045
	Ensemble	0.578±0.042	0.567±0.042	**0.533±0.136**	0.602±0.103	**0.637±0.056**
**FeatureSet**	**Model**	**ACC**	**BACC**	**SEN**	**SPE**	**rocAUC**
MO+MS	AdaBoost	0.675±0.054	0.604±0.078	0.375±0.156	0.833±0.028	0.663±0.074
	DT	0.641±0.051	0.601±0.060	0.475±0.100	0.728±0.043	0.599±0.061
	LDA	0.690±0.055	0.629±0.075	0.433±0.140	0.825±0.026	0.693±0.100
	LogR	0.646±0.056	0.623±0.075	0.550±0.162	0.697±0.066	0.677±0.097
	RF	**0.693±0.050**	0.611±0.075	0.350±0.171	**0.873±0.056**	0.684±0.085
	SVM	0.675±0.041	0.618±0.083	0.433±0.224	0.802±0.061	0.640±0.124
	XGB	0.667±0.041	0.611±0.050	0.433±0.181	0.789±0.119	0.666±0.084
	Ensemble	0.664±0.058	**0.645±0.071**	**0.583±0.159**	0.706±0.085	**0.703±0.103***

FeatureSet	Model	ACC	BACC	SEN	SPE	rocAUC
MO+GT	AdaBoost	0.690±0.010	0.627±0.038	0.425±0.136	0.829±0.062	0.666±0.058
	DT	0.652±0.057	0.624±0.058	0.533±0.126	0.714±0.096	0.633±0.059
	LDA	0.632±0.074	0.587±0.066	0.442±0.086	0.732±0.107	0.626±0.057
	LogR	0.649±0.025	**0.628±0.029**	0.558±0.113	0.697±0.077	0.669±0.034
	RF	**0.693±0.028**	0.623±0.025	0.400±0.048	**0.846±0.050**	0.691±0.075
	SVM	0.646±0.031	0.594±0.023	0.425±0.090	0.763±0.082	0.645±0.054
	XGB	0.638±0.050	0.585±0.050	0.417±0.102	0.754±0.081	0.656±0.058
	Ensemble	0.626±0.043	0.614±0.045	**0.575±0.068**	0.653±0.050	**0.694±0.064**
**FeatureSet**	**Model**	**ACC**	**BACC**	**SEN**	**SPE**	**rocAUC**
MS+GT	AdaBoost	**0.661±0.041**	0.583±0.047	0.333±0.121	**0.833±0.077**	**0.676±0.043**
	DT	0.581±0.044	0.540±0.027	0.408±0.035	0.671±0.084	0.553±0.048
	LDA	0.632±0.056	0.597±0.062	0.483±0.113	0.710±0.070	0.606±0.080
	LogR	0.629±0.039	0.589±0.060	0.458±0.132	0.719±0.026	0.602±0.091
	RF	0.638±0.043	0.570±0.052	0.350±0.096	0.789±0.035	0.632±0.061
	SVM	0.621±0.041	0.588±0.037	0.483±0.076	0.693±0.072	0.617±0.055
	XGB	0.635±0.045	0.595±0.053	0.467±0.112	0.724±0.063	0.630±0.058
	Ensemble	0.618±0.053	**0.616±0.030**	**0.608±0.149***	0.623±0.147	0.669±0.064
**FeatureSet**	**Model**	**ACC**	**BACC**	**SEN**	**SPE**	**rocAUC**
MO+MS+GT	AdaBoost	0.647±0.041	0.572±0.042	0.333±0.098	0.811±0.076	0.656±0.056
	DT	0.594±0.078	0.552±0.081	0.417±0.121	0.688±0.089	0.548±0.070
	LDA	0.629±0.075	0.591±0.068	0.467±0.068	0.715±0.099	0.606±0.076
	LogR	0.635±0.029	0.617±0.025	0.558±0.105	0.675±0.083	0.657±0.033
	RF	**0.681±0.039**	0.603±0.049	0.350±0.116	**0.855±0.063**	0.671±0.047
	SVM	0.641±0.040	0.601±0.033	0.475±0.056	0.728±0.069	0.668±0.046
	XGB	0.649±0.051	0.592±0.043	0.408±0.068	0.777±0.087	0.663±0.051
	Ensemble	0.649±0.035	**0.634±0.046**	**0.583±0.106**	0.684±0.052	**0.685±0.048**

Note: All results are reported as mean±standard deviation, computed from NestedCV.
Within each feature set, the best-performing model is **bolded**. The overall best result (across all models and feature sets) is both **bolded** and marked with an asterisk *** (bolded*)**.

Supplementary Table S4_D || Performance comparison of base models and ensemble using individual and combination of feature sets for the LCDP task.

FeatureSet	Model	MAE	MSE	RMSE	R^2
MO	AdaBoost	**0.396 ± 0.072***	0.596 ± 0.296	0.751 ± 0.198	0.081 ± 0.148
	DT	0.564 ± 0.231	1.152 ± 0.878	1.013 ± 0.395	-0.694 ± 0.778
	GPR	0.429 ± 0.122	0.626 ± 0.346	0.764 ± 0.231	0.063 ± 0.189
	RF	0.455 ± 0.128	0.642 ± 0.357	0.777 ± 0.219	0.027 ± 0.123
	Ridge	0.474 ± 0.089	0.616 ± 0.335	0.758 ± 0.227	0.082 ± 0.155
	SVR	0.416 ± 0.106	0.627 ± 0.373	0.760 ± 0.248	0.083 ± 0.202
	XGB	0.508 ± 0.118	0.742 ± 0.389	0.836 ± 0.232	-0.119 ± 0.057
	Ensemble	0.407 ± 0.096	**0.531 ± 0.307***	**0.701 ± 0.221***	**0.212 ± 0.177***
**FeatureSet**	**Model**	**MAE**	**MSE**	**RMSE**	**R^2**
MS	AdaBoost	0.484 ± 0.088	0.700 ± 0.276	0.821 ± 0.178	-0.128 ± 0.269
	DT	0.514 ± 0.113	0.845 ± 0.374	0.899 ± 0.211	-0.488 ± 0.934
	GPR	0.502 ± 0.127	0.621 ± 0.269	0.774 ± 0.163	-0.039 ± 0.337
	RF	0.500 ± 0.123	0.744 ± 0.323	0.845 ± 0.194	-0.182 ± 0.243
	Ridge	0.472 ± 0.102	0.662 ± 0.334	0.790 ± 0.215	-0.004 ± 0.066
	SVR	**0.434 ± 0.118**	0.725 ± 0.406	0.821 ± 0.251	-0.071 ± 0.140
	XGB	0.449 ± 0.091	0.666 ± 0.318	0.796 ± 0.200	-0.036 ± 0.156
	Ensemble	0.496 ± 0.125	**0.577 ± 0.243**	**0.747 ± 0.156**	**0.054 ± 0.234**
**FeatureSet**	**Model**	**MAE**	**MSE**	**RMSE**	**R^2**
GT	AdaBoost	0.448 ± 0.102	0.727 ± 0.359	0.830 ± 0.221	-0.110 ± 0.095
	DT	0.659 ± 0.249	1.541 ± 1.087	1.171 ± 0.462	-2.076 ± 3.423
	GPR	**0.419 ± 0.114**	0.743 ± 0.368	0.839 ± 0.221	-0.136 ± 0.059
	RF	0.524 ± 0.088	0.741 ± 0.321	0.845 ± 0.184	-0.183 ± 0.154
	Ridge	0.572 ± 0.098	0.831 ± 0.399	0.891 ± 0.219	-0.305 ± 0.219
	SVR	0.428 ± 0.109	0.708 ± 0.351	0.819 ± 0.219	-0.079 ± 0.078
	XGB	0.530 ± 0.165	0.865 ± 0.490	0.898 ± 0.271	-0.300 ± 0.286
	Ensemble	0.476 ± 0.142	**0.643 ± 0.318**	**0.781 ± 0.204**	**0.015 ± 0.027**
**FeatureSet**	**Model**	**MAE**	**MSE**	**RMSE**	**R^2**
MO+MS	AdaBoost	**0.399 ± 0.092**	0.600 ± 0.356	0.745 ± 0.236	0.116 ± 0.167
	DT	0.506 ± 0.193	0.880 ± 0.591	0.890 ± 0.333	-0.235 ± 0.332
	GPR	0.433 ± 0.104	0.668 ± 0.422	0.781 ± 0.269	0.038 ± 0.206
	RF	0.452 ± 0.125	0.590 ± 0.361	0.742 ± 0.221	0.109 ± 0.157
	Ridge	0.474 ± 0.095	0.621 ± 0.342	0.760 ± 0.233	0.079 ± 0.174
	SVR	0.415 ± 0.111	0.614 ± 0.370	0.751 ± 0.249	0.105 ± 0.216
	XGB	0.470 ± 0.085	0.660 ± 0.337	0.788 ± 0.222	0.001 ± 0.161

FeatureSet	Model	MAE	MSE	RMSE	R^2
	**Ensemble**	0.410 ± 0.102	**0.538 ± 0.341**	**0.704 ± 0.230**	**0.207 ± 0.178**
**FeatureSet**	**Model**	**MAE**	**MSE**	**RMSE**	**R^2**
**MO+GT**	**AdaBoost**	**0.400 ± 0.077**	0.619 ± 0.278	0.770 ± 0.181	0.019 ± 0.190
	**DT**	0.614 ± 0.249	1.539 ± 1.152	1.155 ± 0.505	-1.958 ± 3.586
	**GPR**	0.419 ± 0.114	0.743 ± 0.368	0.839 ± 0.221	-0.136 ± 0.059
	**RF**	0.471 ± 0.080	0.632 ± 0.287	0.778 ± 0.184	0.006 ± 0.134
	**Ridge**	0.528 ± 0.082	0.699 ± 0.337	0.814 ± 0.213	-0.074 ± 0.133
	**SVR**	0.421 ± 0.113	0.690 ± 0.356	0.806 ± 0.227	-0.041 ± 0.113
	**XGB**	0.455 ± 0.050	0.636 ± 0.234	0.786 ± 0.155	-0.036 ± 0.207
	**Ensemble**	0.425 ± 0.073	**0.558 ± 0.279**	**0.725 ± 0.200**	**0.141 ± 0.203**
**FeatureSet**	**Model**	**MAE**	**MSE**	**RMSE**	**R^2**
**MS+GT**	**AdaBoost**	0.470 ± 0.114	0.767 ± 0.402	0.850 ± 0.234	-0.164 ± 0.093
	**DT**	0.598 ± 0.208	1.331 ± 0.898	1.096 ± 0.403	-1.586 ± 2.909
	**GPR**	**0.419 ± 0.114**	0.743 ± 0.368	0.839 ± 0.221	-0.136 ± 0.059
	**RF**	0.521 ± 0.094	0.725 ± 0.324	0.835 ± 0.188	-0.151 ± 0.142
	**Ridge**	0.568 ± 0.100	0.820 ± 0.402	0.883 ± 0.224	-0.280 ± 0.218
	**SVR**	0.428 ± 0.109	0.709 ± 0.352	0.819 ± 0.219	-0.080 ± 0.078
	**XGB**	0.546 ± 0.128	0.799 ± 0.363	0.872 ± 0.218	-0.245 ± 0.216
	**Ensemble**	0.476 ± 0.136	**0.641 ± 0.318**	**0.780 ± 0.203**	**0.018 ± 0.025**
**FeatureSet**	**Model**	**MAE**	**MSE**	**RMSE**	**R^2**
**MO+MS+GT**	**AdaBoost**	**0.410 ± 0.075**	0.609 ± 0.304	0.760 ± 0.198	0.063 ± 0.111
	**DT**	0.584 ± 0.155	1.232 ± 0.744	1.060 ± 0.366	-1.136 ± 1.537
	**GPR**	0.429 ± 0.107	0.730 ± 0.386	0.828 ± 0.238	-0.097 ± 0.141
	**RF**	0.518 ± 0.149	0.822 ± 0.677	0.858 ± 0.330	-0.185 ± 0.420
	**Ridge**	0.529 ± 0.086	0.697 ± 0.340	0.813 ± 0.215	-0.069 ± 0.134
	**SVR**	0.421 ± 0.113	0.690 ± 0.357	0.806 ± 0.227	-0.041 ± 0.114
	**XGB**	0.492 ± 0.083	0.673 ± 0.303	0.801 ± 0.196	-0.054 ± 0.181
	**Ensemble**	0.428 ± 0.076	**0.559 ± 0.295**	**0.724 ± 0.207**	**0.155 ± 0.142**

Note: All results are reported as mean±standard deviation, computed from NestedCV.
Within each feature set, the best-performing model is **bolded**. The overall best result (across all models and feature sets) is both **bolded** and marked with an asterisk *** (bolded*)**.

Supplementary Table S5_A || Top 20 important features identified using XAI-based methods for CN—ADD classification task.

Sr.	Ensemble Feature Importance		SHAP Feature Importance		LIME Feature Importance	
	Feature	Importance	Feature	Importance	Feature	Importance
1	MO_SV_Left-Hippocampus	0.1154	MO_SV_Left-Hippocampus	0.0636	MO_SV_Left-Hippocampus	0.1912
2	MO_CT_lh_entorhinal	0.0794	Amyloid_Status	0.0616	MO_CT_rh_parsopercularis	0.1628
3	MO_SV_Left-Amygdala	0.0789	MO_CT_rh_parsopercularis	0.0514	MO_SV_Left-Putamen	0.1196
4	Amyloid_Status	0.0648	MO_SV_Left-Putamen	0.0410	MO_CT_lh_bankssts	0.1089
5	MO_CT_rh_parsorbitalis	0.0547	MO_CT_lh_bankssts	0.0383	MO_CT_lh_superiorfrontal	0.0897
6	MO_CT_rh_entorhinal	0.0505	MO_CT_lh_superiorfrontal	0.0325	MO_SV_CC_Central	0.0763
7	MS_TBSS_WMmaskFA	0.0478	MO_CT_rh_lateraloccipital	0.0274	Amyloid_Status	0.0748
8	MO_CT_lh_superiortemporal	0.0471	MO_SV_CC_Central	0.0266	MO_CT_rh_caudalanteriorcingulate	0.0729
9	MO_SV_Right-Hippocampus	0.0444	MO_CT_rh_caudalanteriorcingulate	0.0252	MO_CT_rh_inferiortemporal	0.0562
10	MO_SV_Left-Accumbens-area	0.0385	MO_CT_rh_inferiortemporal	0.0226	MO_CT_rh_lateralorbitofrontal	0.0372
11	MO_SV_Right-Pallidum	0.0368	MO_CT_lh_parahippocampal	0.0202	MS_RH_meanMD	0.0292
12	MO_CT_rh_lateralorbitofrontal	0.0357	MO_CT_rh_lingual	0.0183	MO_CT_rh_paracentral	0.0234
13	MO_SV_3rd-Ventricle	0.0336	MO_CT_lh_supramarginal	0.0169	MO_CT_lh_middletemporal	0.0201
14	MS_mean_MD	0.0300	MO_CT_lh_superiortemporal	0.0169	MO_CT_rh_inferiorparietal	0.0197
15	MO_CT_lh_parsopercularis	0.0291	MO_SV_Right-Amygdala	0.0165	MO_SV_Left-Thalamus	0.0178
16	MO_SV_BPV	0.0283	MO_CT_rh_entorhinal	0.0160	MO_CT_rh_lateraloccipital	0.0169
17	MO_SV_Right-Lateral-Ventricle	0.0280	MO_CT_lh_middletemporal	0.0150	MO_CT_rh_temporalpole	0.0160
18	MO_CT_lh_temporalpole	0.0273	MO_SV_Left-Lateral-Ventricle	0.0149	MO_SV_Right-Thalamus	0.0160
19	MO_SV_Left-Lateral-Ventricle	0.0269	MO_CT_lh_caudalanteriorcingulate	0.0146	MO_CT_rh_entorhinal	0.0157
20	MO_CT_rh_bankssts	0.0262	MS_TBSS_WMmaskFA	0.0145	MO_CT_lh_caudalanteriorcingulate	0.0147

Supplementary Table S5_B || Top 20 important features identified using XAI-based methods for MCI—ADD classification task.

Sr.	Ensemble Feature Importance		SHAP Feature Importance		LIME Feature Importance	
	Feature	Importance	Feature	Importance	Feature	Importance
1	MO_SV_Left-Hippocampus	0.0623	MO_SV_Right-Putamen	0.8237	MO_SV_Right-Putamen	0.1218
2	Amyloid_Status	0.0539	MO_CT_lh_fusiform	0.6147	MO_CT_lh_isthmuscingulate	0.1137
3	MO_SV_Left-Amygdala	0.0429	MO_SV_Left-Thalamus	0.5700	MO_CT_lh_fusiform	0.1092
4	MO_SV_Right-Caudate	0.0339	MO_CT_lh_isthmuscingulate	0.5327	MO_SV_Left-Thalamus	0.0943

5	MO_CT_lh_parsopercularis	0.0303	Amyloid_Status	0.5182	MO_CT_rh_parahippocampal	0.0820
6	MO_CT_rh_entorhinal	0.0299	MO_CT_rh_lateralorbitofrontal	0.4906	MO_CT_lh_caudalmiddlefrontal	0.0653
7	MO_CT_rh_parahippocampal	0.0291	MO_SV_Right-Thalamus	0.4705	MO_SV_CC_Central	0.0646
8	MO_CT_rh_parsorbitalis	0.0283	MO_CT_lh_parstriangularis	0.3658	MO_CT_lh_superiorparietal	0.0591
9	MO_SV_Left-Thalamus	0.0248	MO_CT_rh_parahippocampal	0.3615	MO_CT_rh_superiortemporal	0.0561
10	MO_CT_lh_superiortemporal	0.0243	MO_CT_lh_superiorparietal	0.3561	MO_CT_rh_posteriorcingulate	0.0487
11	MO_CT_rh_medialorbitofrontal	0.0232	MO_CT_lh_precentral	0.3502	MO_SV_CC_Mid_Anterior	0.0456
12	MO_SV_BPV	0.0214	MO_CT_rh_posteriorcingulate	0.3438	MO_SV_Right-Thalamus	0.0423
13	MO_CT_lh_parahippocampal	0.0193	MO_CT_rh_supramarginal	0.2972	MO_CT_lh_rostralmiddlefrontal	0.0378
14	MO_CT_lh_caudalanteriorcingulate	0.0181	MO_CT_lh_posteriorcingulate	0.2822	MO_SV_Left-Hippocampus	0.0369
15	MO_SV_Right-Putamen	0.0164	MO_CT_lh_temporalpole	0.2615	MO_CT_rh_inferiorparietal	0.0359
16	MO_CT_rh_temporalpole	0.0160	MO_CT_rh_pericalcarine	0.2610	MO_CT_rh_parstriangularis	0.0351
17	MO_CT_rh_bankssts	0.0159	MO_SV_BPV	0.2564	MO_SV_Right-Accumbens-area	0.0313
18	MO_SV_Right-Amygdala	0.0153	MO_CT_lh_rostralmiddlefrontal	0.2505	MO_CT_lh_precentral	0.0294
19	MO_SV_3rd-Ventricle	0.0148	MO_CT_lh_caudalmiddlefrontal	0.2410	MO_SV_BPV	0.0294
20	MO_SV_Right-Hippocampus	0.0146	MO_SV_CC_Mid_Anterior	0.2362	MO_CT_lh_parstriangularis	0.0284

Supplementary Table S5_C || Top 20 important features identified using XAI-based methods for CN—MCI classification task.

Sr.	Ensemble Feature Importance		SHAP Feature Importance		LIME Feature Importance	
	**Feature**	**Importance**	**Feature**	**Importance**	**Feature**	**Importance**
1	MS_mean_MD	0.0208	MO_SV_Left-Accumbens-area	0.0234	MO_SV_Left-Accumbens-area	0.1203
2	MO_SV_Left-Accumbens-area	0.0175	MO_CT_rh_middletemporal	0.0193	MO_SV_CC_Posterior	0.0883
3	MO_CT_rh_lateraloccipital	0.0175	Amyloid_Status	0.0191	MO_CT_rh_lingual	0.0838
4	MO_CT_rh_pericalcarine	0.0125	MO_CT_rh_lingual	0.0190	MO_CT_lh_caudalmiddlefrontal	0.0836
5	MO_CT_lh_precentral	0.0121	MO_CT_lh_temporalpole	0.0180	MO_CT_lh_medialorbitofrontal	0.0747
6	MO_CT_lh_entorhinal	0.0120	MO_SV_BPV	0.0178	MO_CT_rh_transversetemporal	0.0711
7	MO_CT_rh_insula	0.0120	MO_CT_lh_caudalmiddlefrontal	0.0166	MO_SV_Right-Thalamus	0.0707
8	MO_SV_3rd-Ventricle	0.0119	MO_CT_rh_transversetemporal	0.0155	MO_SV_CC_Mid_Posterior	0.0700
9	MO_CT_lh_superiorfrontal	0.0112	MO_SV_CC_Posterior	0.0153	MO_SV_CC_Central	0.0674
10	MO_SV_Left-Thalamus	0.0102	MO_SV_CC_Central	0.0148	MO_SV_Left-Hippocampus	0.0662
11	MO_CT_lh_isthmuscingulate	0.0099	MO_SV_CC_Mid_Posterior	0.0147	MO_CT_rh_paracentral	0.0581
12	MO_SV_Right-Hippocampus	0.0098	MO_CT_rh_precuneus	0.0139	MO_CT_lh_postcentral	0.0569

13	MO_CT_lh_cuneus	0.0095	MO_CT_lh_supramarginal	0.0137	MO_SV_Right-Putamen	0.0550
14	MO_SV_Left-Amygdala	0.0093	MO_CT_rh_lateraloccipital	0.0135	MO_SV_Right-Hippocampus	0.0544
15	MO_CT_lh_caudalmiddlefrontal	0.0090	MO_CT_lh_fusiform	0.0133	Amyloid_Status	0.0543
16	MO_CT_lh_parstriangularis	0.0088	MO_CT_rh_entorhinal	0.0132	MO_CT_rh_precuneus	0.0540
17	Amyloid_Status	0.0087	MO_CT_lh_medialorbitofrontal	0.0131	MO_CT_lh_superiorparietal	0.0501
18	MO_SV_Left-Hippocampus	0.0087	MO_SV_Right-Thalamus	0.0131	MO_CT_lh_pericalcarine	0.0453
19	MO_SV_Right-Pallidum	0.0085	MO_CT_lh_lingual	0.0130	MO_CT_rh_bankssts	0.0420
20	MO_CT_rh_caudalmiddlefrontal	0.0084	MO_SV_Right-Accumbens-area	0.0129	MO_CT_rh_parsopercularis	0.0399

Supplementary Table S5_D || Top 20 important features identified using XAI-based methods for LCDP task.

Left	Ensemble Feature Importance		SHAP Feature Importance		LIME Feature Importance	
	Feature	Importance	Feature	Importance	Feature	Importance
1	MO_CT_rh_entorhinal	0.0046	MO_CT_rh_parsorbitalis	0.0495	MO_SV_CC_Mid_Anterior	0.4065
2	MO_CT_rh_bankssts	0.0034	MO_CT_rh_inferiorparietal	0.0456	MO_CT_lh_middletemporal	0.0987
3	MO_CT_rh_fusiform	0.0033	MO_CT_lh_frontalpole	0.0424	MO_CT_lh_insula	0.0833
4	MO_CT_lh_fusiform	0.0032	MO_CT_rh_rostralmiddlefrontal	0.0421	MO_CT_rh_caudalmiddlefrontal	0.0823
5	MO_SV_Right-Amygdala	0.0027	MO_CT_rh_superiortemporal	0.0417	MO_CT_lh_superiorparietal	0.0769
6	MO_CT_rh_isthmuscingulate	0.0025	MO_CT_rh_caudalmiddlefrontal	0.0408	MO_CT_rh_caudalanteriorcingulate	0.0738
7	MO_CT_rh_middletemporal	0.0023	MO_SV_Right-Hippocampus	0.0403	MO_SV_Left-Caudate	0.0733
8	MO_CT_lh_precuneus	0.0022	MO_CT_lh_middletemporal	0.0403	Amyloid_Status	0.0728
9	MO_SV_Right-Hippocampus	0.0021	MO_CT_lh_inferiortemporal	0.0378	MO_CT_lh_isthmuscingulate	0.0699
10	MO_CT_rh_posteriorcingulate	0.0021	MO_CT_rh_isthmuscingulate	0.0370	MO_CT_rh_inferiorparietal	0.0694
11	MO_CT_rh_inferiortemporal	0.0019	MO_CT_rh_lateralorbitofrontal	0.0364	MO_CT_lh_inferiorparietal	0.0679
12	MO_SV_Left-Hippocampus	0.0018	MO_SV_Left-Putamen	0.0327	MO_CT_rh_middletemporal	0.0664
13	MO_CT_rh_rostralanteriorcingulate	0.0016	MO_CT_lh_entorhinal	0.0308	MO_CT_lh_pericalcarine	0.0654
14	MO_CT_lh_inferiortemporal	0.0011	MO_CT_lh_superiorfrontal	0.0299	MO_CT_rh_postcentral	0.0637
15	MO_CT_lh_bankssts	0.0011	MO_CT_rh_lingual	0.0288	MO_CT_lh_superiorfrontal	0.0616
16	MO_SV_Left-Lateral-Ventricle	0.0008	MO_CT_lh_isthmuscingulate	0.0287	MO_CT_lh_parsopercularis	0.0595
17	MO_CT_rh_supramarginal	0.0007	MO_CT_lh_postcentral	0.0287	MO_CT_rh_bankssts	0.0583
18	MO_SV_Left-Accumbens-area	0.0007	MO_SV_Right-Pallidum	0.0268	MO_SV_Right-Putamen	0.0560
19	MO_SV_Left-Amygdala	0.0007	MO_CT_rh_parstriangularis	0.0256	MO_SV_Left-Accumbens-area	0.0541
20	MO_CT_rh_caudalanteriorcingulate	0.0006	MO_SV_Left-Amygdala	0.0254	MO_CT_rh_lateraloccipital	0.0531

Supplementary Figure S1 || Receiver operating characteristic (ROC) curves for disease stage classification (DSC) across different MRI-derived feature sets. ROC curves represent mean out-of-fold (OOF) performance across cross-validation folds for ensemble models trained using individual and combined feature sets. Curves were generated using pooled OOF prediction scores, with 95% confidence intervals estimated via bootstrap resampling (1000 iterations). Shaded regions indicate uncertainty across folds, and ROC-AUC values correspond to those reported in Supplementary Tables S4—S6.

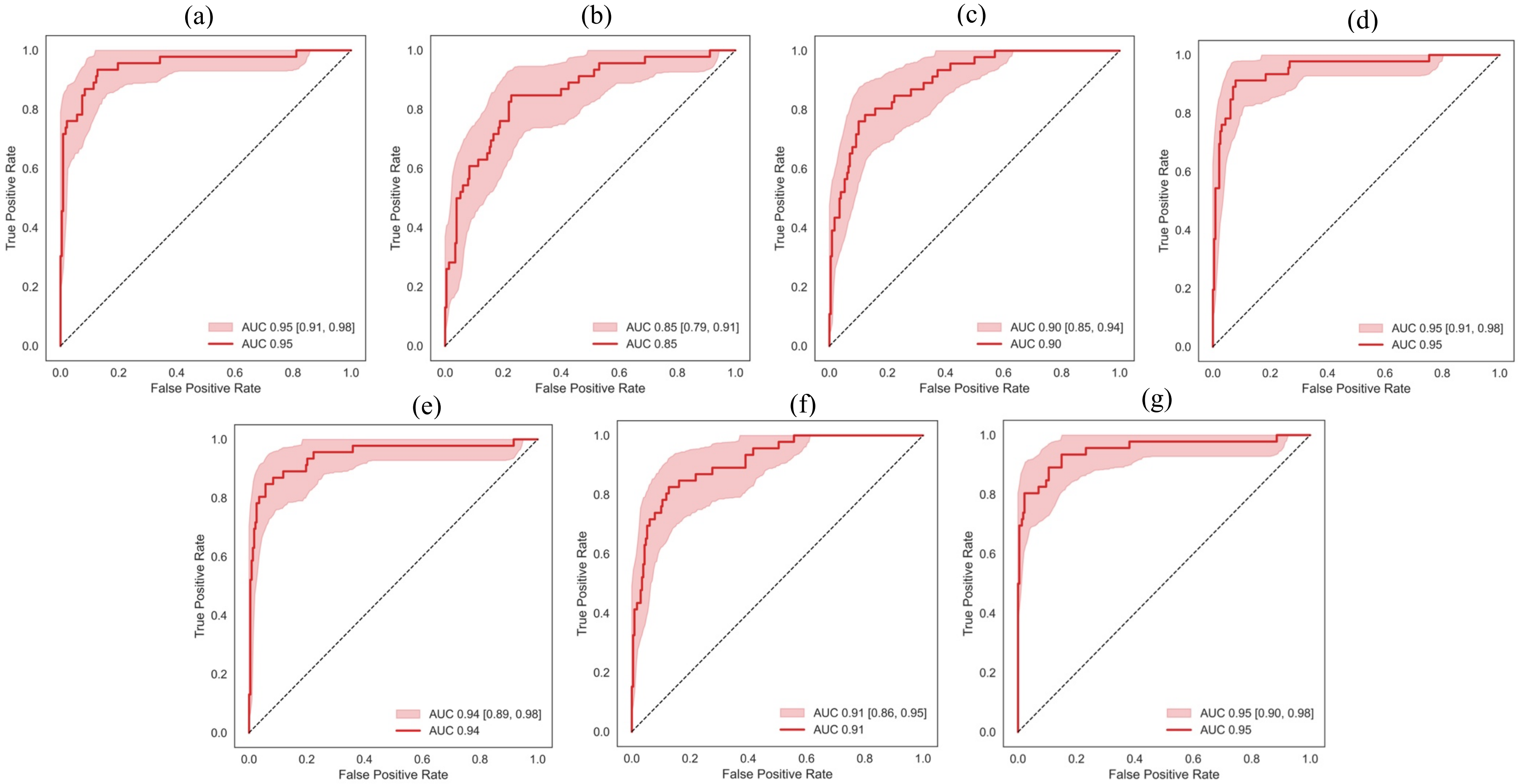

Figure S1_A. ROC curves for ensemble models trained using different MRI-derived feature sets for the CN—ADD classification task: (a) MO; (b) MS; (c) GT; (d) MO+MS; (e) MO+GT; (f) MS+GT; and (g) MO+MS+GT.

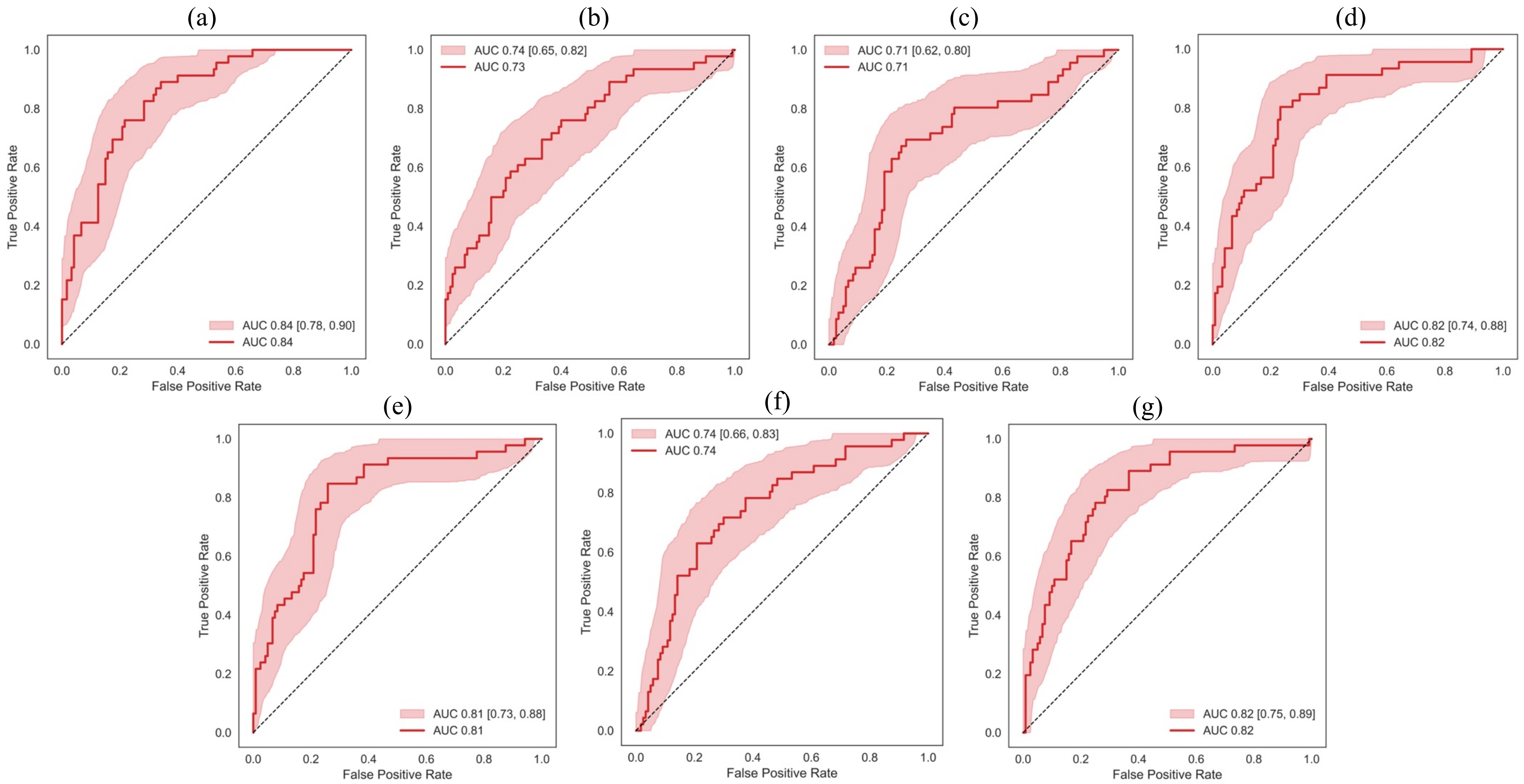

Figure S1_B. ROC curves for ensemble models trained using different MRI-derived feature sets for the MCI—ADD classification task: (a) MO; (b) MS; (c) GT; (d) MO+MS; (e) MO+GT; (f) MS+GT; and (g) MO+MS+GT.

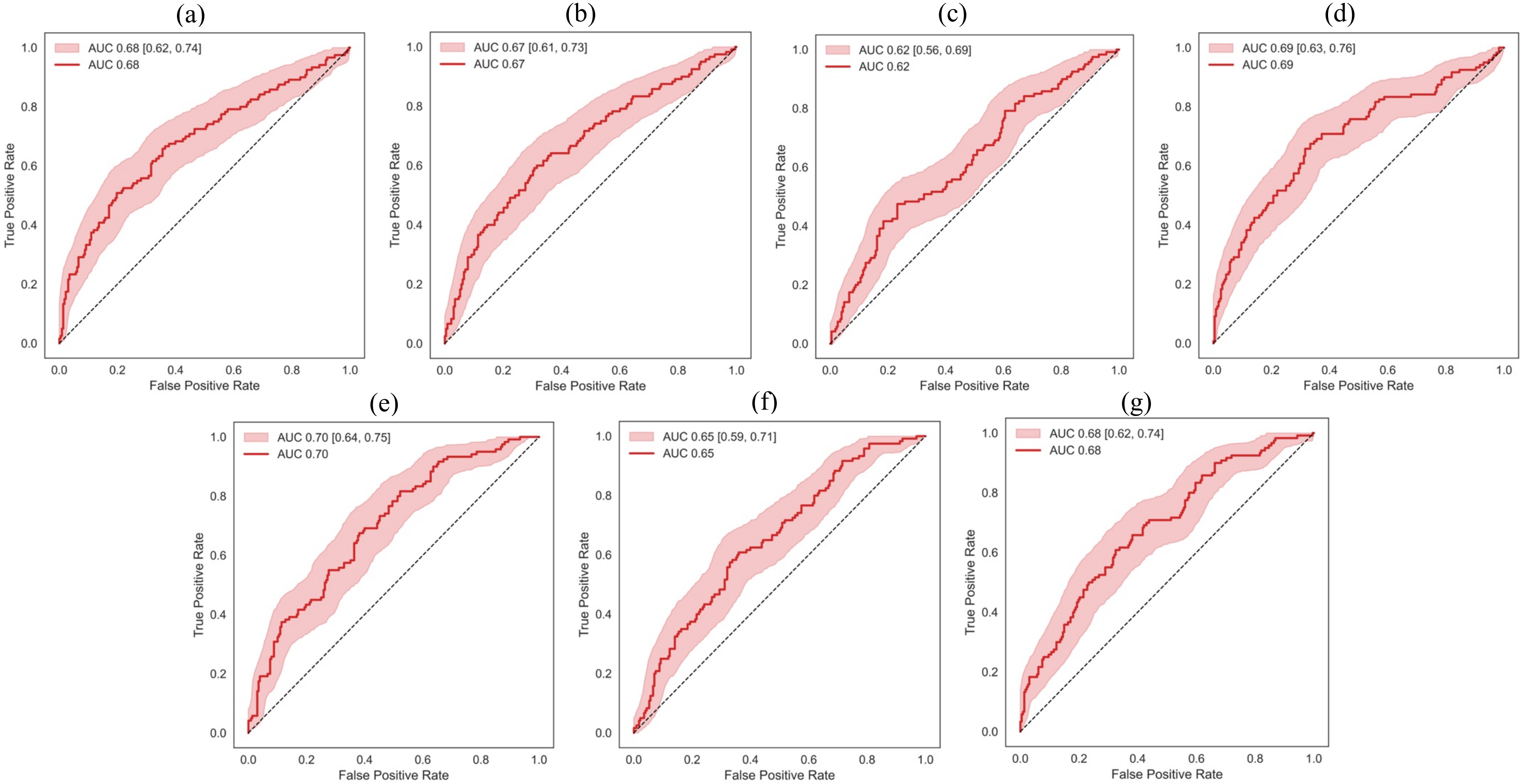

Figure S1_C. ROC curves for ensemble models trained using different MRI-derived feature sets for the CN—MCI classification task: (a) MO; (b) MS; (c) GT; (d) MO+MS; (e) MO+GT; (f) MS+GT; and (g) MO+MS+GT.
